# Projecting the economic burden of chronic kidney disease at the patient level (*Inside CKD*): a microsimulation modelling study

**DOI:** 10.1016/j.eclinm.2024.102615

**Published:** 2024-05-02

**Authors:** Steven Chadban, Mustafa Arıcı, Albert Power, Mai-Szu Wu, Francesco Saverio Mennini, José Javier Arango Álvarez, Juan Jose Garcia Sanchez, Salvatore Barone, Joshua Card-Gowers, Alexander Martin, Lise Retat

**Affiliations:** a Royal Prince Alfred Hospital and University of Sydney, Camperdown, NSW, 2050, Australia; b Division of Nephrology, Department of Internal Medicine, Hacettepe University, Ankara, 06230, Türkiye; c North Bristol NHS Trust, Southmead Hospital, Bristol, BS10 5NB, UK; d Department of Internal Medicine, School of Medicine, College of Medicine, Taipei Medical University, Taipei City, 110007, Taiwan; e Universidad del Quindío, Quindío, Colombia; f Economic Evaluation and HTA-CEIS, Department of Economics and Finance, Faculty of Economics, University of Rome “Tor Vergata”, Rome, 00133, Italy; g Global Health Economics, BioPharmaceuticals, AstraZeneca, Academy House, 136 Hills Road, Cambridge, CB2 8PA, UK; h Global Medical Affairs, BioPharmaceuticals, AstraZeneca, Gaithersburg, MD, 20878, USA; i HealthLumen Limited, London, EC3N 2PJ, UK

**Keywords:** Burden of disease, Chronic kidney disease, Health economics, Microsimulation, Health policy

## Abstract

**Background:**

The growing burden of chronic kidney disease (CKD) places substantial financial pressures on patients, healthcare systems, and society. An understanding of the costs attributed to CKD and kidney replacement therapy (KRT) is essential for evidence-based policy making. *Inside CKD* maps and projects the economic burden of CKD across 31 countries/regions from 2022 to 2027.

**Methods:**

A microsimulation model was developed that generated virtual populations using national demographics, relevant literature, and renal registries for the 31 countries/regions included. Patient-level country/region-specific cost data were extracted via a pragmatic local literature review and under advisement from local experts. Direct cost projections were generated for diagnosed CKD (by age, stage 3a–5), KRT (by modality), cardiovascular complications (heart failure, myocardial infarction, stroke), and comorbidities (hypertension, type 2 diabetes).

**Findings:**

For the 31 countries/regions, *Inside CKD* projected that annual direct costs (US$) of diagnosed CKD and KRT would increase by 9.3% between 2022 and 2027, from $372.0 billion to $406.7 billion. Annual KRT-associated costs were projected to increase by 10.0% from $169.6 billion to $186.6 billion between 2022 and 2027. By 2027, patients receiving KRT are projected to constitute 5.3% of the diagnosed CKD population but contribute 45.9% of the total costs.

**Interpretation:**

The economic burden of CKD is projected to increase from 2022 to 2027. KRT contributes disproportionately to this burden. Earlier diagnosis and proactive management could slow disease progression, potentially alleviating the substantial costs associated with later CKD stages. Data presented here can be used to inform healthcare resource allocation and shape future policy.

**Funding:**

10.13039/100004325AstraZeneca.

**Research in context**

**Evidence before this study**

A pragmatic literature review was independently performed by local agencies in 31 countries/regions, which collected patient-level annual direct cost data and definitions for chronic kidney disease (CKD) stages 3a–5; kidney replacement therapy (KRT)—haemodialysis, peritoneal dialysis, and kidney transplantation initiation and maintenance; disease-associated comorbidities of hypertension and type 2 diabetes; cardiovascular (CV) complications of heart failure, myocardial infarction, and stroke. Local language search terms and exact databases were not recorded but the search was performed under the direction of local experts who formed the Scientific Steering Committee for *Inside CKD,* and data were obtained from available national registries, published literature, and healthcare databases. It is apparent that a comprehensive understanding of the economic burden of CKD is crucial for raising disease awareness, as well as for policymakers, healthcare providers and other stakeholders to allocate resources effectively, develop sustainable healthcare systems, and implement preventive strategies. The quality of comprehensive cost data is poor due to a paucity of studies, variable costing definitions, and differences in practice patterns and health system structures globally. The economic burden of CKD, and particularly kidney failure, contributes to substantial proportions of annual health spending, and existing global models (e.g. the Global Burden of Disease Study) provide snapshots of only the epidemiological burden.

**Added value of this study**

We produced an analysis of the economic burden of CKD across 31 participating countries/regions, and project that between 2022 and 2027 annual direct healthcare costs of CKD (pre-KRT) will increase by 8.7% from ∼US$202 billion to ∼US$220 billion for the entire data set. By 2027, costs of KRT are projected to be ∼US$187 billion, despite these patients representing a small proportion (5.3%) of the diagnosed CKD population. CKD and KRT contribute to a substantial proportion of overall national healthcare expenditures, projected to range from 1.6% to 26.6% in 2027. *Inside CKD* represents the first analysis of its kind to map and project the economic burden of CKD on a global scale, generating cost projections refined by age, disease stage, comorbidity status, CV complication, and/or KRT modality.

**Implications of all the available evidence**

These country-specific and global projections of direct costs will help to raise awareness of the burden of CKD, inform evidence-based policies, and enable cost-effective resource allocation to address the anticipated economic impact of CKD. The microsimulation can be programmed to examine the cost-effectiveness of interventions such as screening for CKD, and findings could be used to inform strategies for slowing CKD progression to reduce the financial burden of kidney failure and KRTs.

## Introduction

Chronic kidney disease (CKD) is associated with significant healthcare resource use and costs. Later disease stages (4–5) incur a higher per-patient cost compared with earlier disease stages (1–3 b).^1^^,^^2^ CKD progression can be slowed with renin-angiotensin-aldosterone system inhibitors or sodium–glucose co-transporter 2 inhibitors, but the risk of disease progression, requirement for kidney replacement therapy (KRT), risk of cardiovascular (CV) complications, and risk of mortality remains.^3^ The costs associated with KRT contribute significantly to the economic burden of CKD, although patients requiring these treatments constitute a small proportion of the total CKD population. Despite this, the exact burden remains unquantified in many countries, and disease awareness is low, which precludes effective evidence-based resource allocation.^4^^,^^5^

The substantial burden of kidney failure affects all nations, regardless of demographics, income, or health infrastructure. In the UK, the per-patient costs of KRT are up to six times greater than the overall healthcare costs associated with earlier stages of CKD.^6^ In the USA, the costs attributed to kidney failure in 2017 accounted for 7.2% of total Medicare fee-for-service expenditure.^7^ In upper–middle income countries, KRT has a significant impact on expenditure; in Türkiye, for example, costs in 2000 corresponded to approximately 5.5% of the country's total health expenditure.^8^ The high financial burden of kidney failure requiring KRT results in health inequities in the resources available to tackle the burden of CKD. For example, government-funded care for CKD without KRT is available in 40% of high-income countries compared with 13% of low-income countries.^9^ Service availability, capacity, and willingness to pay for KRTs vary globally, but are often insufficient to meet demand; by 2030, 14.5 million individuals are estimated to have kidney failure requiring KRT, but only 5.4 million individuals will receive treatment owing to economic, social, and political factors.^9^

Owing to the substantial unmet need for KRT, and its disproportionately higher per-patient cost compared with earlier disease stages, strategies are needed to reduce the burden of CKD, such as screening to detect early-stage disease. Such strategies should consider the complex aetiology of CKD, which is driven by factors including the rising prevalence of non-communicable diseases (hypertension, type 2 diabetes mellitus [T2D], and vascular disease), as well as an ageing population, climate change, and lifestyle choices.^4^^,^^10^ Understanding global prevalence, overlaid with temporal trends, may provide opportunities for targeted interventions that benefit patients and healthcare systems. Despite the need to engage with this complexity, there is a lack of granular data on the impact of disease stage, age, or comorbidity status on the financial burden of CKD. Estimates of the national economic burden are often incomplete, historic, or lacking entirely, particularly in lower–middle-income countries.

*Inside CKD* is the first comprehensive, validated microsimulation to map and project the global economic burden of CKD by age, disease stage, comorbidity (hypertension and/or T2D), and CV complication status (heart failure, myocardial infarction, or stroke). A comprehensive overview of the clinical impact of CKD carried out by the *Inside CKD* microsimulation has been published alongside this manuscript, which projected a high CKD and KRT burden in 2027.^11^ Here, we report the direct economic burden associated with diagnosed CKD in 2022 and 2027 for 31 countries/regions.

## Methods

### Study design

A methodology, detailing the technical development of the microsimulation and its clinical parametrisation, has been previously described.^12^ Further methodological information for the economic parameters and a detailed description of the associated data inputs has also been previously described.^1^

**Fig. 1:**
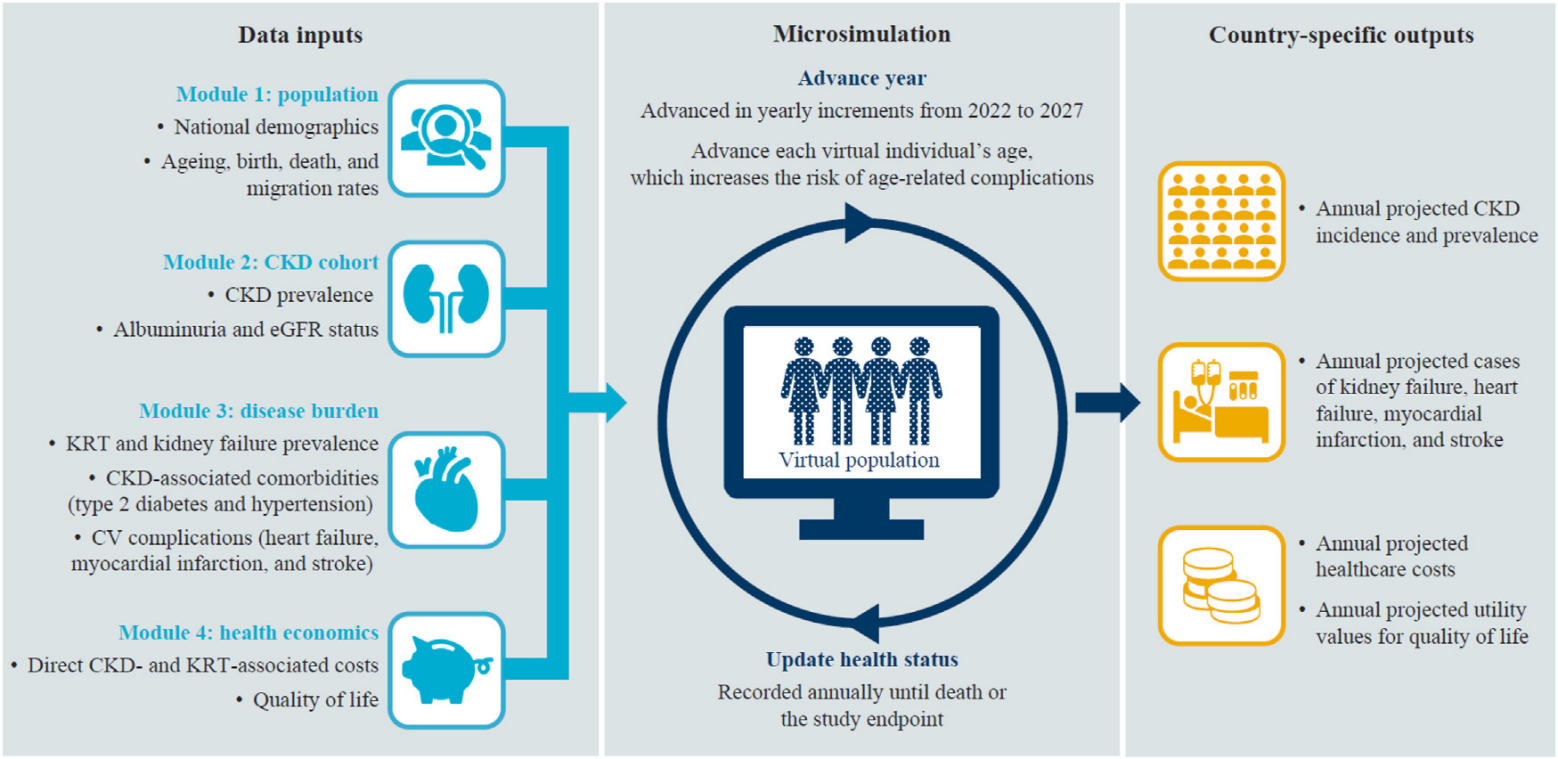
Overview of the structure of the Inside CKD microsimulation. Image adapted from the Inside CKD methodology manuscript published by Tangri and colleagues.^12^ CKD, chronic kidney disease; CV, cardiovascular; eGFR, estimated glomerular filtration rate; KRT, kidney replacement therapy.

The *Inside CKD* microsimulation was developed to project the global burden of CKD for a population of virtual individuals generated using national demographic data and relevant, country-specific publications reporting clinical and economic disease-related data (Fig. 1).

This study was conducted in accordance with ethical principles of the Declaration of Helsinki and Good Clinical Practice guidelines. This study did not require informed consent or institutional/ethical review board approval because this is a non-interventional study based on secondary data use.

### Overview

The microsimulation was designed to reflect current real-world clinical progression and outcomes. A series of data input modules generated a hypothetical cohort that was reflective of a given national population. These modules included a CKD cohort with associated disease burden (hypertension and T2D as comorbidities; heart failure, myocardial infarction, and stroke as CV complications) and a health economics module (Fig. 1). Virtual individuals progressed through the microsimulation in yearly increments over an adjustable time horizon; in the simulation reported here, the time horizon is from 2022 to 2027. Annually, an individual's age was updated, and their health status was amended in line with national demographics and associated descriptive statistics, until death or study endpoint. For example, ageing increased the relative risk of complications, all-cause mortality, and the likelihood of a new CKD diagnosis or progression of existing disease. The default perspective of the analysis was the local healthcare system, although data inputs varied and healthcare systems vary globally, so outputs may not be directly comparable. To determine rates of CKD progression, individual clinical characteristics were used alongside regression analyses based on the DISCOVER CKD cohort—a multi-country non-interventional study that investigated patient characteristics, clinical management, practice patterns, and disease progression for patients with CKD.^13^ These inputs generated estimated glomerular filtration rate slopes to determine disease progression. These slopes were assumed to be consistent across countries/regions.

Here, we report the results for the economic analyses of 31 middle and high income countries/regions: Australia, Belgium, Brazil, Canada, China, Colombia, Denmark, France, Germany, Greece, Hungary, India (cost inputs for India were disaggregated and comprised private, public and charity healthcare costs), Israel, Italy, Japan, Mexico, Netherlands, Philippines, Poland, Romania, Saudi Arabia, Singapore, South Korea, Spain, Sweden, Taiwan, Thailand, Türkiye, UAE (Emirati and expatriate populations were modelled separately. UAE expatriate data are provided in the Supplementary Tables only), UK, and USA (Medicare and commercial care systems were modelled). The selection of participating countries was based on a strategic approach that considered factors such as willingness to participate, including a requirement for support from one or more local clinical experts that formed the Scientific Steering Committee. The Scientific Steering Committee comprised local clinical experts who provided advice on the modelling approach, conceptualisation, clinical assumptions, validation of data inputs, addressing of data gaps, model calibrations, and interpretation of results from a global and local perspective (Table S1).

### Health economics data inputs

Input data for each country/region were sourced from a pragmatic literature review that considered varied sources including published literature, local databases, and national registries. The full list of health economic data inputs is provided in the Supplementary section in the publication by Jha and colleagues.^1^ Per-patient direct costs were calculated for each country/region (i.e. cost per case, per year). These included costs associated with diagnosed CKD stages 3a–5; KRT costs (haemodialysis, peritoneal dialysis, kidney transplantation surgery, and maintenance costs for those living with a kidney transplant); and costs associated with CV complications (heart failure, myocardial infarction, and stroke). Costs for CKD stages 1 and 2 were assumed to be zero because most cases remain undiagnosed. This approach was taken in attempt to produce conservative cost projections. Direct costs were calculated using the cost inputs and based on prevalence projections for CKD, KRT, and associated complications and/or comorbidities. Prevalence results are reported separately and within this volume by Chertow and colleagues.^11^ The costs of CKD stages 3a–5 excluded hospitalisation costs, when possible, to mitigate confounding effects from comorbidities and complications.

All costs reported were adjusted for inflation and provided in 2022 US$ after correcting for purchasing power parity.^1^ Cost definitions and data availability varied, therefore comparisons between countries should be interpreted carefully; treatment costs could only be accounted for if reported in the literature landscape for a given location. When country-specific input data were unavailable, a multi-dimensional algorithm was used to identify suitable proxy data, which considered relatability of economy and epidemiology (Supplementary Methods, Table S2). The Scientific Steering Committee assessed these data and advised on suitability. Indirect costs were not included in the microsimulation owing to a paucity of input data.

### Health economics outputs

Individual-level costs were calculated based on an individual health/treatment status. These were used to generate population-level model outputs and were calculated for each country/region and year, which included: projected direct costs of diagnosed CKD by stage, age, and comorbidity status (hypertension, T2D); projected directs costs of KRT by modality (haemodialysis, peritoneal dialysis, and kidney transplantation incidence and maintenance); and projected direct costs of CV complications (heart failure, myocardial infarction, or stroke) in the diagnosed CKD population. Any means reported here are unweighted and were derived from the sum (totals) for each participating country/region regardless of population size.

### Model validation

Model validation is described in detail by Tangri and colleagues.^12^ The microsimulation was validated from five perspectives: face validity, internal validity, external validity, predictive validity, and cross-validity. External validation for individual countries/regions was performed for the economic analyses by comparing microsimulation outputs for CKD and/or KRT costs with real-world data that did not form inputs for this microsimulation.

### Role of the funding source

The funder of the study was involved in the study design, data analysis, data interpretation and writing of the report. The funder had no role in data collection, which were conducted by HealthLumen. Authors (LR, AM and JC-G) checked and corroborated the data. All authors had full access to all data and the corresponding author had final responsibility for the decision to submit for publication.

## Results

### Overall costs: diagnosed CKD and KRT

**Fig. 2:**
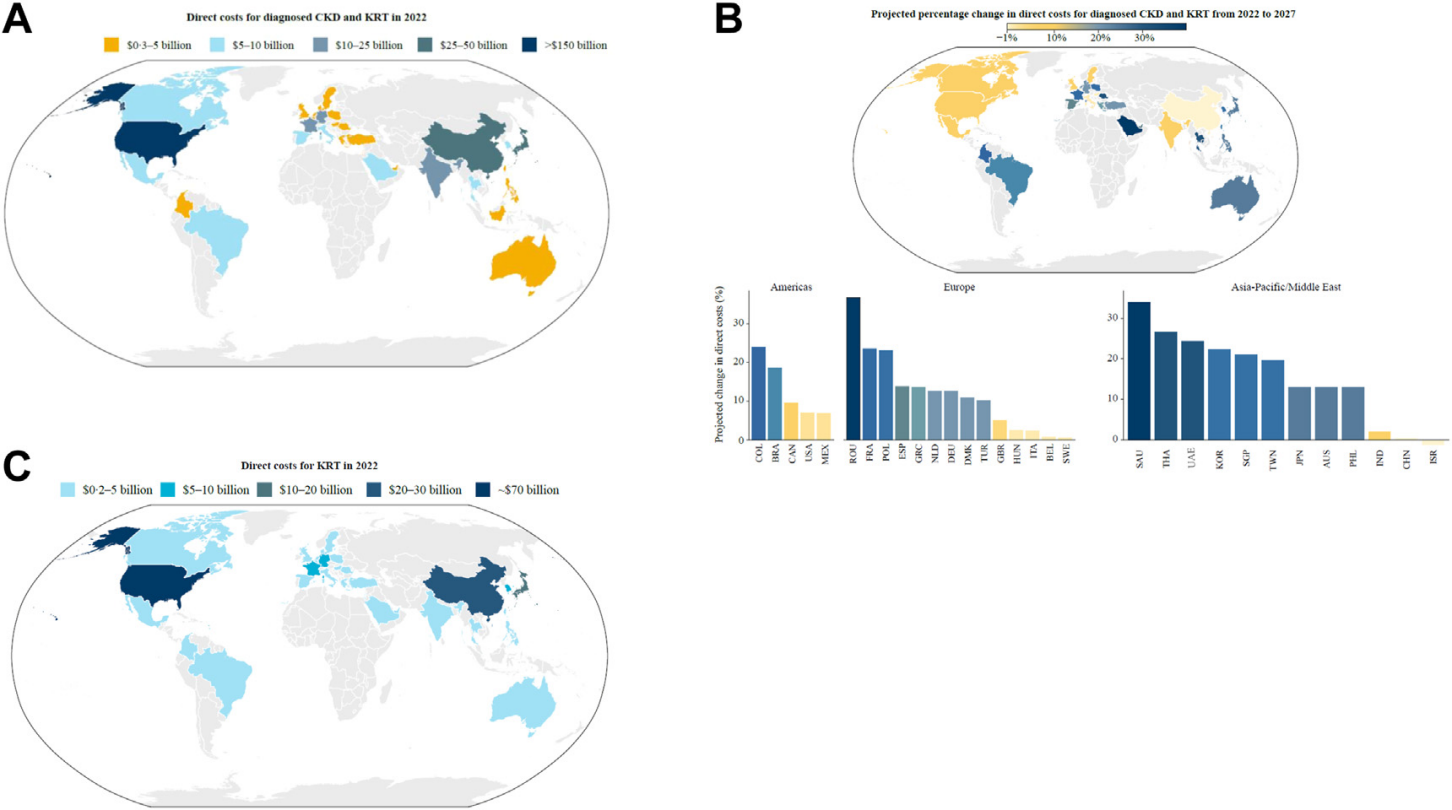
Projected costs of CKD and KRT in 2022. **(A)** Projected total direct costs for diagnosed CKD and KRT combined (numerical values can be found in Table S3). **(B)** Projected percentage change in the total direct costs of diagnosed CKD and KRT from 2022 to 2027 (numerical values can be found in Table S3). **(C)** Direct costs for KRT in 2022 (numerical values stratified by modality can be found in Table S6). Note: The UAE has a large and diverse expatriate population with a different CKD profile; only the Emirati population has been presented here. For Israel, total CKD and KRT costs are projected to decrease by 1.16%, from $1.55 billion in 2022 to $1.53 billion in 2027. The decrease is driven by a projected decrease in CKD stage 4 costs. Cost increases are projected for CKD stage 3a, CKD stage 5, haemodialysis, peritoneal dialysis and kidney transplant incidence and maintenance. CKD, chronic kidney disease; KRT, kidney replacement therapy.

Across all 31 countries/regions, the total direct costs of CKD and KRT were projected to increase by 9.3% between 2022 and 2027, from $372.0 billion to $406.7 billion (Fig. 2A and B, Table S3), with all countries showing an increase in absolute costs except for Israel (projected at $1.55 billion in 2022, and $1.53 billion in 2027. Table S3). Of the above total, USA costs (Medicare and commercial) were projected to make the largest overall contribution of any given country/region and were projected to be $161.9 billion in 2022 and $173.4 billion in 2027.

### Overall costs: diagnosed CKD (pre-KRT)

Across all 31 countries/regions, CKD costs (pre-KRT) were projected to rise by 8.7% between 2022 and 2027, from $202.4 billion in 2022 to $220.1 billion in 2027 (2027 range: $0.06 billion–$99.5 billion. Table S4).

When stratified by age, the largest CKD (pre-KRT) costs were observed in those aged 65 years and over, totalling $153.2 billion by 2027 (Table S5).

It is notable that costing definitions varied, and inter-country comparisons should be considered with care. For example, Poland demonstrated very low CKD stage 3a–5 (pre-KRT) costs and projected an absolute decrease from $0.07 billion in 2022 to $0.06 billion in 2027, but these costs comprised physician visits and diagnostic tests only and may be underrepresented compared with other *Inside CKD* countries/regions.

### Overall costs: KRT

Across all 31 countries/regions, KRT costs were projected to increase by 10.0% from 2022 to 2027. In 2022 they were projected at $169.6 billion, which represented 45.6% of the total CKD and KRT costs (Fig. 2C, Table S6). By 2027, this was projected to be $186.6 billion (range: $0.26 billion–$73.90 billion), which represented 45.9% of total CKD and KRT costs (Table S6). Despite these high-cost contributions, *Inside CKD* projected that patients receiving KRT comprised only 5.3% of the diagnosed CKD population (data not shown).

Within KRT modalities, haemodialysis represented the largest overall cost burden, which was projected to be $133.7 billion in 2022 and $146.8 billion in 2027 (Table S6).

### National healthcare expenditure

**Fig. 3:**
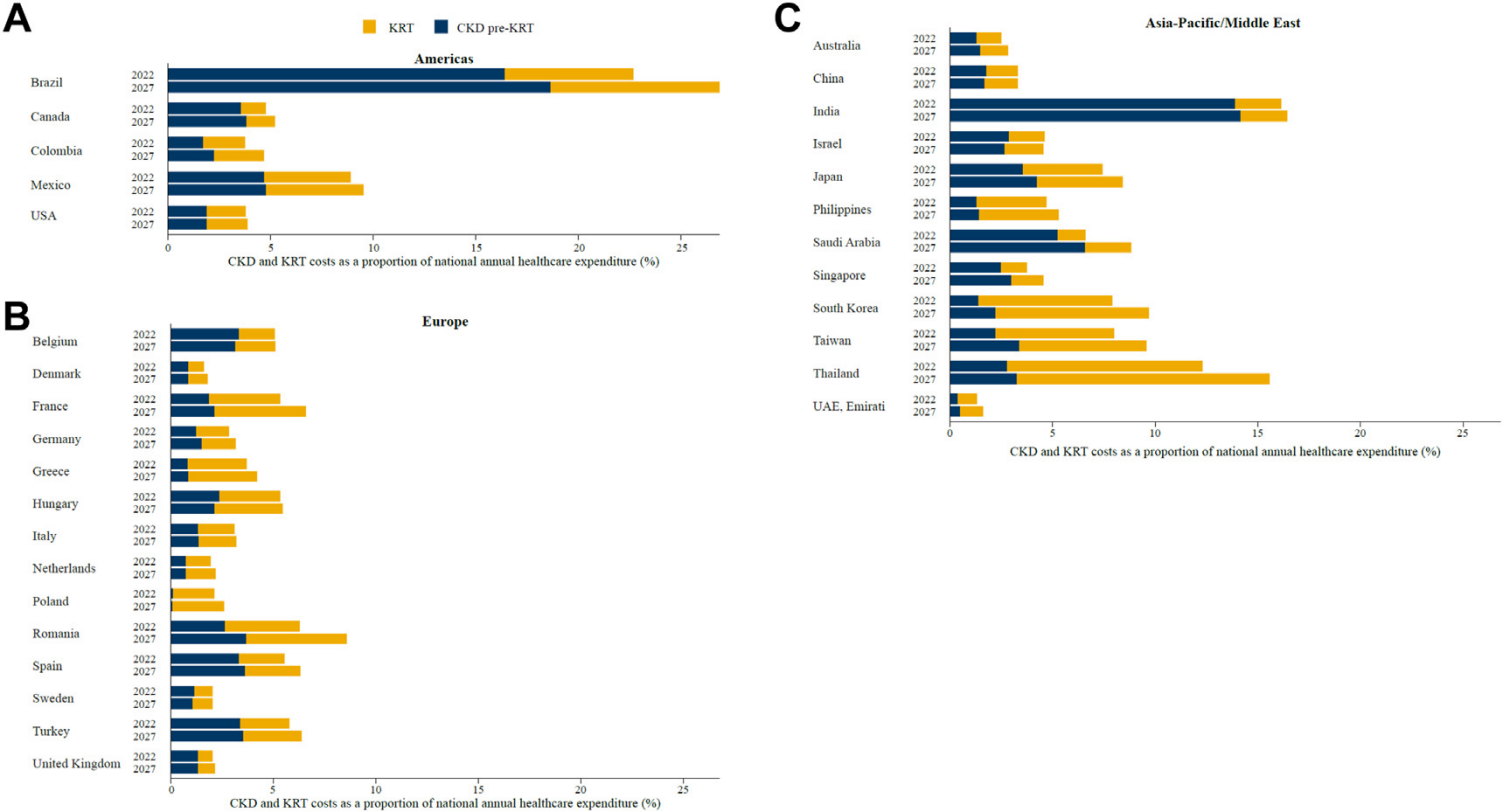
Projected direct CKD and KRT costs as a proportion of national annual healthcare expenditure (%) in 2022 and 2027. Numerical values can be found in Table S7. Note: The UAE has a large and diverse expatriate population with a different CKD profile; only the Emirati population has been presented here. CKD, chronic kidney disease; KRT, kidney replacement therapy.

In terms of national healthcare budgets, CKD and KRT were projected to account for an increasing proportion of healthcare expenditure, from a mean of 5.6% in 2022 to a projected mean of 6.4% in 2027 (Fig. 3, Table S7). Haemodialysis consistently contributed the most to costs out of the three KRT modalities (Table S7), reflecting the higher number of patients receiving haemodialysis compared with other treatments.

### CV complications in the diagnosed CKD population

CV complications within the diagnosed CKD population constituted a substantial economic burden over the analysis period for all 31 countries/regions (Table 1). By 2027, total annual costs for CV complications were projected to be $108.5 billion for heart failure, $155.2 billion for myocardial infarction, and $90.0 billion for stroke.

**Table 1:** Projected direct costs of cardiovascular complications stratified by heart failure, myocardial infarction, or stroke in the population diagnosed with CKD in 2027.

Country/region	Projected 2027 costs, US$ billion		
	Heart failure	Myocardial infarction	Stroke
**Americas**			
Brazil	1.98	7.91	0.17
Canada	2.03	1.68	1.86
Colombia	0.64	9.58	0.22
Mexico	2.45	28.59	0.86
USA^a^	51.43	13.68	44.76
**Europe**			
Belgium	0.72	0.86	0.57
Denmark	0.18	0.67	0.30
France	1.39	1.03	3.86
Germany	0.15	0.21	0.40
Greece	0.45	0.50	0.27
Hungary	0.02	0.05	0.12
Italy	3.29	3.72	2.48
Netherlands	0.47	0.45	0.28
Poland	1.76	2.53	5.23
Romania	0.23	0.26	0.59
Spain	2.67	2.00	1.31
Sweden	0.27	0.08	0.25
Türkiye	0.40	0.10	0.67
UK	0.74	1.25	1.07
**Asia–Pacific/Middle East**			
Australia	2.00	0.85	0.05
China	8.00	21.61	6.51
India^a^	7.64	7.86	8.49
Israel	0.06	0.19	0.21
Japan	15.89	44.89	6.55
Philippines	1.15	0.53	0.66
Saudi Arabia	1.24	1.50	0.46
Singapore	0.14	0.50	0.27
South Korea	0.04	0.58	0.81
Taiwan	0.06	0.04	0.06
Thailand	0.92	1.42	0.60
UAE Emirati^b^	0.11	0.10	0.02
**Total**	108.5	155.2	90.0

CKD, chronic kidney disease.

a In settings without a single public healthcare system but with distinct input data for each aspect of funding, the model was adapted to use a commercial or equivalent framework, or a mixed funding model. In the case of the USA, costs were split into Medicare and commercial categories; for India, costs were split into four components: charitable, private, employment insurance and public.

b The UAE has a large and diverse expatriate population with a different CKD profile; only the Emirati population has been presented here.

### Comorbidities associated with the diagnosed CKD population

Projected costs associated with comorbidities in the diagnosed CKD population remained a considerable economic burden over the analysis period for all 31 countries/regions, totalling $144.5 billion in 2027 (Table 2). This included $95.7 billion for CKD with hypertension, $20.7 billion for CKD with T2D, and $28.1 billion for CKD with hypertension and T2D.

**Table 2:** Projected direct costs of comorbidities among the population with diagnosed CKD stratified by those with hypertension and/or type 2 diabetes mellitus in 2027.

Country/region	Projected 2027 costs, US$ billion		
	Hypertension	Type 2 diabetes	Hypertension and type 2 diabetes
**Americas**			
Brazil	4.43	0.51	1.75
Canada	1.93	0.97	0.91
Colombia	0.60	0.07	0.21
Mexico	1.64	0.24	0.86
USA^a^	38.27	10.61	11.74
**Europe**			
Belgium	0.90	0.13	0.22
Denmark	0.13	0.02	0.03
France	3.00	0.40	0.69
Germany	3.30	0.48	0.78
Greece	0.16	0.02	0.04
Hungary	0.18	0.03	0.06
Italy	1.47	0.17	0.56
Netherlands	0.33	0.05	0.08
Poland	0.03	0.004	0.01
Romania	0.41	0.06	0.14
Spain	2.61	0.30	0.98
Sweden	0.31	0.06	0.11
Turkey	1.39	0.21	0.34
UK	1.50	0.21	0.36
**Asia–Pacific/Middle East**			
Australia	0.70	0.11	0.17
China	11.74	1.73	2.78
India^a^	5.23	1.54	1.47
Israel	0.32	0.08	0.06
Japan	9.63	1.43	2.30
Philippines	0.30	0.04	0.08
Saudi Arabia	2.44	0.70	0.71
Singapore	0.43	0.06	0.10
South Korea	0.98	0.14	0.24
Taiwan	0.49	0.19	0.19
Thailand	0.82	0.12	0.20
UAE Emirati^b^	0.05	0.01	0.01
**Total**	95.7	20.7	28.1

CKD, chronic kidney disease.

a In settings without a single public healthcare system but with distinct input data for each aspect of funding, the model was adapted to use a commercial or equivalent framework, or a mixed funding model. In the case of the USA, costs were split into Medicare and commercial categories; for India, costs were split into four components: charitable, private, employment insurance and public.

b The UAE has a large and diverse expatriate population with a different CKD profile; only the Emirati population has been presented here.

### External validation

Validation of outputs has been described previously, and a UK example is used to describe the process in detail, which comprised four aspects: external validation, internal validation, cross-validation, and face validity.^11^^,^^12^ This was conducted in accordance with the International Society for Pharmacoeconomics and Outcomes Research and the Society for Medical Decision Making (ISPOR-SMDM) task force. Costs were validated against external literature sources of real-world events that did not form data inputs (Table S8), which involved consideration by the Scientific Steering Committee (face validity). It is notable that data sources for external validation varied greatly in terms of endpoints, data collection years and methodology, and cost definitions, hence direct comparisons are not provided or advised, but the exercise is included for transparency of reporting.

## Discussion

*Inside CKD* is the first analysis of its kind to map and project the economic burden of CKD on a global scale, generating cost projections stratified by age, disease stage, comorbidity status, CV complications, and/or KRT modality. Here, we demonstrate a projected increase in the economic burden of diagnosed CKD for 31 countries/regions between 2022 and 2027. This projected increase is driven by the disproportionate patient-level cost contributions of severe disease (including KRT), which is consistent with published literature.^5^, ^6^, ^7^, ^8^^,^^14^^,^^15^

When examining by age group, the largest direct financial burden was among patients with CKD aged 65 years and over, highlighting the need to support an increasingly ageing population; the burden among those aged 65 years and over was projected at $153.2 billion, out of $219.9 billion across all age groups by 2027. Improved life expectancy, driven in part by socioeconomic progress, has resulted in a growing proportion of elderly people. This socioeconomic progress supports increased access to healthcare, advances in medication, and the increased availability of screening programmes for diseases such as hypertension and T2D, all of which are combined with improvements in preventive medicine.^16^^,^^17^ Ageing is associated with a natural decline of kidney function, coinciding with an increased risk of non-communicable diseases, including T2D and hypertension, with the net effect being a rising prevalence of CKD. Ageing is also associated with conditions such as arthritis for which treatment may cause or exacerbate CKD. Approximately one in five patients with CKD will receive nephrotoxic medications for other conditions, increasing their risk of CKD progression.^18^^,^^19^ Increasing age is also associated with risk of acute kidney injury, an important antecedent of CKD.

In this microsimulation, the direct financial impact of CKD on patients of working age may have been proportionately lower than for older age groups, but still amounted to billions of dollars across the data set (projected to be $66.7 billion for those aged 18–64 years by 2027). Although beyond the remit of this study, the indirect economic burden incurred from CKD in younger patients (aged 18–64 years) is an important consideration. Employment and productivity may vary, but individuals with later stages of CKD are often less economically productive, leading to a substantial accrual of lost income, indirect costs, and, early retirement.^6^^,^^20^ The Spanish Society of Nephrology demonstrated that, in 2009, gainful employment was 33.3% lower for patients receiving KRT than for the age-matched general population.^20^ Studies such as these, which require primary data collection and qualitative data analyses, are scarce but provide insights into the significant social and personal impact of the disease on working-age individuals with CKD. In some localities, the impact of a reduced capacity for employment and productivity is compounded by the need to cover healthcare costs. For example, in India, up to 50% of dialysis patients are self-paying.^21^ Indirect costs measure the broader, societal effect of CKD and are worthy of consideration, but because these costs are complex to quantify, they are often not included or are acknowledged to be conservatively calculated and limited to discrete events such as excess hospital stays.^6^ *Inside CKD* examined direct costs, so we may assume that the true burden is greater than projected.

Given the cost burden and the trend towards an increasing prevalence, CKD seems under-appreciated relative to other chronic diseases. This under-appreciation is reflected in research priorities; for example, the funding from the US National Institutes of Health for kidney research was approximately half that of diabetes in 2022, despite a similar prevalence ($0.67 billion versus $1.17 billion, respectively).^22^ Patient awareness is important for treatment engagement and effective delivery of care, but is demonstrably low for CKD.^5^^,^^23^ This may reflect insufficient resources, or a lack of commitment to education around self-management. Clinical inertia may translate into a lack of disease recognition and diagnosis, which is reflected in current statistics on underdiagnosis.^11^^,^^24^ The Kidney Disease: Improving Global Outcomes (KDIGO) 2022 Clinical Practice Guidelines emphasise that a holistic approach to disease management is required. Ideally, this would take into account the complex cardiorenal interplay between CKD and other chronic conditions, including T2D or hypertension, ensuring patients are incorporated into a single pathway for treatment and risk management of these comorbid conditions.^25^

As multi-directional, pathogenetic relationships exist between CKD, T2D, and hypertension, strategies aimed at reducing one comorbidity may indirectly affect another, and targeted approaches may bring population-wide benefits. The multimodal approach of *Inside CKD* permitted the examination of the burden of cardiorenal complications and projected that the economic burden of hypertension and/or T2D within the diagnosed CKD population will increase,^11^ which is consistent with data that demonstrate the number of people aged 30–79 years with hypertension has doubled from 1990 to 2019.^26^ It should be noted that while comorbid conditions (and cardiorenal complications) were costed for separately in the microsimulation, when input data were unable to be adjusted for multiple comorbidities, some values may be overestimated. The cost of treating individuals with multiple morbidities is not statistically different than the additive cost of treating separate individuals with one of the conditions when age and unrelated health costs are controlled for.^27^ However, as the simulation does not incorporate hospitalisation costs or costs associated with CKD stages 1–2, overall the projection is likely to under-estimate the true economic burden of the disease. The impact of comorbidities and cardiorenal complications may be reduced with earlier diagnosis and timely strategies to slow CKD progression, which have been shown to improve clinical outcomes.^28^ The growing availability of evidence-based interventions that can slow disease progression and reduce associated complications supports the use of proactive treatment, especially given the increased patient-level costs associated with later disease stages.^2^^,^^16^

In this study, direct costs are reported in a global context; however, there was considerable variation in data inputs between countries/regions, necessitating a nuanced interpretation of the results; these variations are discussed in greater detail by Jha and colleagues.^1^ For example, for Australia, CKD stages 3a–5 costs considered ambulatory services, hospitalisation, medication, and medically related consumables, but did not include certain parameters, such as medical imaging, pharmaceuticals, or primary care, and, therefore, may be considered conservative.^1^ For Canada, CKD stages 3a–5 costs considered drug costs, physician visits, emergency department visits, outpatient procedures (including dialysis and other day medicine and surgery procedures), and hospitalisation costs, additionally including comorbidities and complications in patients with CKD.^1^ These data sets should, therefore, be considered in the context of their specific population demographics, national healthcare systems, and local economies. For example, in 2008, the Thai government extended its healthcare coverage to include KRT for treatment-naive patients initiating dialysis and the establishment of new dialysis centres. This potentially high upfront cost may explain the relatively high proportion of national health spending on KRT in Thailand (projected to be 12.2% by 2027).^29^

In terms of how *Inside CKD* compares with other microsimulations, there are no direct cost equivalents; however, data from digital healthcare systems can provide useful insights into current trends. The CaReMe CKD study examined individual data for 2.4 million patients with CKD across 11 countries and estimated the prevalence, key clinical adverse outcomes, and costs of CKD. CKD-related hospital healthcare expenses were identified as a primary driver of costs, exceeding those of heart failure and atherosclerotic CV disease.^30^ These findings are consistent with the *Inside CKD* outputs, which exemplifies how microsimulations can complement real-world data because they allow multiple variables to be incorporated and analysed over multiple years. Microsimulations can thus augment real-world studies, provide critical insights into the future development of national trends, and allow policymakers to assess the financial repercussions of epidemiological data across several years.

Comparison with existing data sets provides one measure for validation, but the overall accuracy of the *Inside CKD* cost projections—as with any microsimulation—inevitably depends on the quality of the input data sources. Although national data were considered the first-choice resource for inputs, when unavailable, proxy data were used. Simulations for some countries/regions required the extensive use of proxies, highlighting a need for real-world epidemiological and financial data that can enhance the overall quality of model inputs in the future. In some cases, there were inconsistencies in the cost definitions between literature sources, which may have undermined the suitability of some input variables; however, when possible, a cost input that was deemed conservative was used to avoid over-projecting costs. With regards to India, a relatively large proportion of its national annual healthcare budget was projected to be spent on pre-KRT CKD-associated costs (13.8% in 2022 and 14.1% in 2027), but cost inputs for India were disaggregated and comprised private, public/state, and charity healthcare costs. Therefore, these costs may not be comparable with other modelled countries for which only public costs were available. Some countries, such as Brazil, have a mixed healthcare system (e.g. public and private); others also involve individually funded and/or charity sponsored care. Comprehensive data covering multiple systems was not often available, which precluded our ability to include uncertainty estimates and may limit the utility of the results and highlights the need for improved data collection.

In terms of other limitations, indirect costs were not within the scope of *Inside CKD*, and, despite the obvious societal impact of kidney failure, there is a lack of available data. The quantitative and qualitative impact of CKD on quality of life, productivity, and employment for a person with CKD or their caregiver could all be usefully explored in future simulations. Hospitalisation costs were excluded from CKD staging costs when possible, owing to the confounding impact of CV disease but these costs are an additional economic burden, meaning that *Inside CKD* projections may be considered conservative. Although the model can be used to examine future trends, its inputs mimic a snapshot of the economic climate from which it was formed, so changes in the cost of living or inflation are not considered. Furthermore, the microsimulation did not account for the effects of the COVID-19 pandemic or its impact on life expectancy. Data show that kidney transplantation activity across 22 countries decreased by 19.1% in 2020 compared with the previous year; this trend may have, in real terms, changed costs relative to our projections and long-term impacts need to be considered.^31^ The effects of climate change on CKD were also not accounted for, and heat stress (caused by extreme heatwaves) has been implicated in the aetiology of CKD and may also indirectly increase costs above our projections.^10^ Further research to examine the impact of these environmental stressors (e.g. pandemics, extreme weather, air quality) would provide useful data for anticipating future trends in increasingly unpredictable times. Renewed data inputs and/or additional modules could be developed within the *Inside CKD* microsimulation to consider these issues and assess their impact on the projections. Additionally, mortality or other health conditions (e.g. renal cancer) could be incorporated to provide a more thorough assessment of the complex CKD aetiology and associated risks.

*Inside CKD* helps to raise awareness of the growing international financial burden of CKD and provides impactful national and region-specific data for policymakers to anticipate the evolving trends of the CKD epidemic. Given the relatively small population size, patients with kidney failure who receive KRT contribute disproportionately to the costs of CKD. Transplantation is the most cost-effective form of KRT, yet capacity is limited by the availability of transplant services and donor kidneys; consequently, the need for dialysis is increasing disproportionately. *Inside CKD* also demonstrated the substantial economic burden among the population with CKD and hypertension, and those aged 65 years and older, thus highlighting the complex disease aetiology of CKD and need to support populations holistically.

The data presented here provide a foundational evidence base for policy makers and health advisors to support the financial planning of health strategies. This in turn may facilitate policies orientated to achieving earlier diagnosis and interventions to mitigate the progressive effects of CKD. Earlier detection through screening and proactive treatment to slow disease progression may result in improved clinical outcomes, reducing the direct and indirect financial burden of CKD, as well as improving patient outcomes and quality of life.

## Contributors

JC-G, AM and LR contributed to study conceptualisation, data curation, formal analysis, investigation, methodology, visualisation, data interpretation, writing concept creation at outline, and writing review and editing of all drafts. SC, MA, AP, M-S W, FSM, and JJAA contributed to study conceptualisation, data curation (review of model inputs and source data), resources, validation, data interpretation, writing concept creation at outline, and writing review and editing of all drafts. JJGS, and SB contributed to overall study supervision, study conceptualisation, formal analysis, methodology, visualisation, data interpretation, writing concept creation at outline, and writing review and editing of all drafts. All authors have access to the underlying study data. Authors SC, JC-G, AM, LR and JJGS verify the underlying study data.

## Data sharing statement

The data sets generated and/or analysed during the current study are available from the corresponding author on reasonable request.

## Declaration of interests

JC-G, AM, and LR are employees of HealthLumen Ltd and AstraZeneca provided funding to HealthLumen Ltd for their contributions to this work. AP, FSM, JJAA, MA, and M-SW have received support from AstraZeneca for their contributions to this work. AP has received payment/honoraria from AstraZeneca for lectures, presentations, advisory boards, and educational events. AP has received payment/honoraria from Bayer for lectures and advisory boards. AP has received payment/honoraria from Boehringer Ingelheim for advisory boards. AP received support from CSL Vifor to attend the European Renal Association–European Dialysis and Transplantation Association congress in 2023. MA has received payment/honoraria from Amgen, Astra Zeneca, Astellas, Bayer, Boehringer Ingelheim, Menarini, MSD, Novo Nordisk, and Sanofi for presentations. MA has received support from AstraZeneca and Astellas for attendance at meetings and/or travel. SC has received payment/honoraria from AstraZeneca, Boehringer Ingelheim, and Bayer for speaker fees and advisory boards. JJGS and SB are employees and shareholders of AstraZeneca. M-SW has received payment/honoraria from AstraZeneca for lectures, presentations, and manuscript writing. M-S W has received payment/honoraria from Baxter, Bayer, GSK, Novartis, Pfizer and Sanofi for lectures and presentations.
