# Supplementary material for: Projecting the economic burden of chronic kidney disease at the patient level (Inside CKD): a microsimulation modelling study
Source: eClinicalMedicine. 2024 May 2;72:102615. doi: 10.1016/j.eclinm.2024.102615 (PMC11247148; doi:10.1016/j.eclinm.2024.102615)
Supplement: Supplementary Tables [file mmc1.pdf]

Projecting the economic burden of chronic kidney disease at the patient level (*Inside CKD*): a microsimulation modelling study

Supplementary materials

Authors

Steven Chadban, Mustafa Arıcı, Albert Power, Mai-Szu Wu, Francesco Saverio Mennini,
José Javier Arango Álvarez, Juan Jose Garcia Sanchez, Salvatore Barone, Joshua Card-Gowers,
Alexander Martin, Lise Retat

Contents

Authors 1
Supplementary methodology 2
Table S1: The *Inside CKD* Scientific Steering Committee 2
Derivation of proxy data if local source data were not available 4
Table S2: Data proxy suggestions by country for economic indicators.[1–3] 4
Supplementary methodology references 7
Supplementary results 8
Table S3: Projected direct costs of diagnosed CKD and KRT in 2022 and 2027 8
Table S4: Projected direct costs of diagnosed CKD (pre-KRT) by CKD stage in 2022 and 2027 10
Table S5: Projected direct costs of diagnosed CKD (pre-KRT) costs by age group in 2022 and 2027 13
Table S6: Projected direct costs of KRT by modality in 2022 and 2027 15
Table S7: CKD and KRT costs as a proportion of national annual healthcare expenditure in 2022 and 2027 17
Table S8: External validation of economic burden of disease outputs. 19
Supplementary results references 24

Supplementary methodology

Table S1: **The *Inside CKD* Scientific Steering Committee**

Region	Key external expert	Affiliation
Australia	Prof. Steven Chadban	Royal Prince Alfred Hospital and University of Sydney, Camperdown, Australia
Belgium	Prof. Michel Jadoul	Cliniques Universitaires Saint-Luc, Université catholique de Louvain, Brussels, Belgium
Brazil	Prof. Marcelo Costa Batista	Hospital Israelita Albert Einstein, São Paulo, Brazil
Canada	Dr Navdeep Tangri	University of Manitoba, Winnipeg, Canada
China	Prof. Guisen Li	Sichuan Academy of Medical Sciences and Sichuan Provincial People's Hospital, Chengdu, China
Colombia	Prof. José Javier Arango Álvarez	Universidad del Quindío, Quindío, Colombia
Denmark	Prof. Christian Fynbo Christiansen	Aarhus University and Aarhus University Hospital
France	Prof. Jean-Michel Halimi	Service de néphrologie-HTA, dialyses et transplantation rénale, CHRU de Tours, Tours, France
Germany	Prof. Kai-Uwe Eckardt	Charité Universitätsmedizin Berlin, Berlin, Germany
Greece	Dr Panos Stafylas	HealThink, Greece
Hungary	Prof. Istvan Wittmann, Dr Boglarka Laczy	University of Pécs, Pécs, Hungary
India	Prof. Vivekanand Jha	The George Institute for Global Health India, New Delhi, India
Israel	Prof. Avraham Karasik	Maccabi Institute for Research and Innovation, Tel Aviv, Israel
Israel	Dr Gil Chernin	Kaplan Medical Center, Faculty of Medicine, Hebrew University of Jerusalem, Jerusalem, Israel
Italy	Prof. Francesco Saverio Mennini	Economic Evaluation and HTA-CEIS, Department of Economics and Finance, Faculty of Economics, University of Rome "Tor Vergata", Rome, Italy
Italy	Prof. Luca De Nicola	Department of Advanced Medical and Surgical Sciences, University of Campania Luigi Vanvitelli, Naples, Italy
Japan	Prof. Eiichiro Kanda	Kawasaki Medical School, Okayama, Japan
Mexico	Prof. José Ricardo Correa-Rotter	Instituto Nacional de Ciencias Médicas y Nutrición Salvador Zubirán, Mexico City, Mexico
Philippines, Singapore, and Thailand	Assoc. Prof. Jason Choo Chon Jun	Singapore General Hospital, Singapore
Poland	Prof. Michał Nowicki	Warsaw University of Technology, Warsaw, Poland
Romania	Prof. Dr Ismail Gener	Fundeni Institute, Bucuresti, Romania
Saudi Arabia	Prof. Saeed M.G Al-Ghamdi	King Abdulaziz University Hospital and King Faisal Specialist Hospital and Research Centre, Jeddah, Saudi Arabia

South Korea	Prof. Kook-Hwan Oh	Seoul National University College of Medicine, Seoul, South Korea
Spain	Prof. Juan Francisco Navarro-González	Research Unit and Nephrology Service, University Hospital Nuestra Señora de Candelaria, Santa Cruz de Tenerife, Spain
Sweden	Prof. Johan Ärnlöv	Department of Neurobiology, Care Sciences and Society, Division of Family Medicine and Primary Care, Karolinska Institute, Stockholm, Sweden
Taiwan	Prof. Mai-Szu Wu	Department of Internal Medicine, School of Medicine, College of Medicine, Taipei Medical University, Taipei City, Taiwan<br><br>Taipei Medical University Research Center of Urology and Kidney, Taipei City, Taiwan<br><br>Taipei Medical University Shuang Ho Hospital, New Taipei City, Taiwan
Türkiye	Prof. Mustafa Arıcı	Division of Nephrology, Department of Internal Medicine, Hacettepe University, Ankara, Türkiye
UAE	Prof. Stephen Holt	SEHA Kidney Care, Abu Dhabi, UAE
UK	Dr Albert Power	North Bristol NHS Trust, Southmead Hospital, Bristol, UK
USA	Prof. Glenn Chertow	Stanford University School of Medicine, California, USA
USA	Prof. Jay Wish	Indiana University School of Medicine, Indianapolis, USA

CEIS=Centre for Economic and International Studies. CHRU=Centre Hospitalier Régional Universitaire. CKD=chronic kidney disease. HTA=health technology assessment. NHS=National Health Service.

Derivation of proxy data if local source data were not available

If suitable local data were not available for one of the countries/regions, we developed a multi-dimensional algorithm using the following process.

- For each country/region, a list of neighbouring countries/regions were generated.
- Key indicators were utilized:
  - Universal Health Coverage index[1]
  - Domestic private health expenditure (% of current health expenditure)[2]
  - Current health expenditure per capita, purchasing power parity (PPP).[3]
- A list of proxy countries/regions were created based on having similar values to the key indictors
- For each of the indicators, a percentage similarity was selected (from ±5 to ±75%) to ensure that a large enough list of countries could be generated.
- If an index country had a 10% prevalence, and we chose ±50%, the list would contain countries with values between 5% and 15% rather than between -40% and 60%.

All data were available through the World Bank Data repository,[4] with the exception of data on overweight and raised blood pressure, which was sourced from the World Health Organization Global Health Observatory.[5]

If data were not available for a proxy selected through this method, then a country that is in close proximity to the target country of interest was be selected and/or advice was sought from external experts (Scientific Steering Committee) about the most appropriate proxy country data to select. External experts were also involved in verifying proxy data and suggesting alternatives to the proxies selected using this method if necessary.

The data were manipulated using Python software. Proxy country suggestions by indicator for each country are shown in the table below.

Table S2: **Data proxy suggestions by country for economic indicators.**[1–3]

	Within 5%	Within 20%	Within 50%	Within 75%
Australia		Canada	Belgium, Canada, Denmark, France, Israel, Italy, Japan, Korea (Republic), Netherlands, Saudi Arabia, Spain, Singapore, Sweden, United Arab Emirates, United Kingdom	Belgium, Canada, Denmark, France, Germany, Greece, Israel, Italy, Japan, Korea (Republic), Netherlands, Poland, Romania, Saudi Arabia, Spain, Singapore, Sweden, United Arab Emirates, United Kingdom
Belgium	Japan	Denmark, France, Germany, Japan, Netherlands, Sweden	Denmark, France, Germany, Japan, Netherlands, Sweden, United Kingdom	Canada, Denmark, France, Germany, Japan, Italy, Netherlands, Sweden, United Arab Emirates, United Kingdom
Brazil			Greece, Mexico,	China, Colombia, Greece, Israel, Korea, Mexico, Poland, Thailand, Türkiye, United Arab Emirates
Canada		Australia	Australia, Belgium, Denmark, France, Germany, Israel, Italy, Japan, Netherlands, Spain, Saudi Arabia, Sweden, United Arab Emirates, United Kingdom	Australia, Belgium, Denmark, France, Germany, Greece, Israel, Italy, Japan, Korea (Republic), Netherlands, Poland, Saudi Arabia Singapore, Spain, Sweden, United Arab Emirates, United Kingdom
China		Colombia	Colombia, Mexico, Thailand, Türkiye	Colombia, Mexico, Romania, Philippines, Thailand, Türkiye
Colombia		China	China, Mexico, Romania, Thailand, Türkiye	China, Mexico, Romania, Thailand, Türkiye

Denmark		Belgium, France, Germany, Japan, Netherlands, Sweden	Belgium, France, Germany, Japan, Netherlands, Sweden, United Kingdom	Belgium, Canada, France, Germany, Italy, Japan, Netherlands, Sweden, United Arab Emirates, United Kingdom
France		Belgium, Denmark, Germany, Japan, Netherlands, Sweden, United Kingdom	Belgium, Denmark, Germany, Italy, Japan, Netherlands, Sweden, United Kingdom	Belgium, Canada, Denmark, Germany, Italy, Japan, Netherlands, Spain, Sweden, United Arab Emirates, United Kingdom
Germany		Belgium, Denmark, France, Japan, Sweden	Belgium, Denmark, France, Netherlands, Japan, Sweden, United Kingdom	Belgium, Canada, Denmark, France, Italy, Netherlands, Japan, Sweden, United Kingdom
Greece		Korea (Republic)	Israel, Korea (Republic), Poland, Romania, Saudi Arabia, Spain	Brazil, China, Colombia, Israel, Italy, Korea (Republic), Mexico, Poland, Romania, Saudi Arabia, Spain, Thailand, Türkiye, United Arab Emirates
India			Philippines	Philippines
Israel		Korea (Republic), Saudi Arabia, Spain	Greece, Italy, Korea (Republic), Poland, Saudi Arabia, Singapore, Spain, United Arab Emirates, United Kingdom	Australia, Belgium, Canada, China, Colombia, France, Greece, Italy, Japan, Korea (Republic), Mexico, Poland, Romania, Saudi Arabia, Singapore, Spain, Türkiye, United Arab Emirates, United Kingdom
Italy		Spain	Australia, Belgium, Canada, Denmark, France, Israel, Japan, Poland, Saudi Arabia, Spain, United Arab Emirates, United Kingdom	Australia, Belgium, Canada, Denmark, France, Germany, Greece, Israel, Japan, Korea (Republic), Netherlands, Poland, Romania, Saudi Arabia, Spain, Sweden, Türkiye, United Arab Emirates, United Kingdom
Japan	Belgium	Belgium, Denmark, France, Germany, Netherlands, Spain	Belgium, Denmark, France, Germany, Netherlands, Spain, Sweden, United Kingdom	Belgium, Canada, Denmark, France, Germany, Italy, Netherlands, Romania, Spain, Sweden, United Arab Emirates, United Kingdom
Korea (Republic)		Greece, Israel	Greece, Israel, Italy, Poland, Saudi Arabia, Spain, United Arab Emirates	Australia, Belgium, Brazil, Canada, China, Colombia, Greece, Israel, Italy, Japan, Mexico, Poland, Romania, Saudi Arabia, Singapore, Spain, Türkiye, United Arab Emirates, United Kingdom

Mexico			China, Colombia,	China, Colombia, Philippines, Romania, Thailand, Türkiye
Netherlands		Belgium, Denmark, France, Germany, Japan, Sweden	Belgium, Canada, Denmark, France, Germany, Italy, Japan, Sweden, United Kingdom	Australia, Belgium, Canada, Denmark, France, Germany, Italy, Japan, Poland, Saudi Arabia, Spain, Sweden, United Arab Emirates, United Kingdom
Philippines			India	India
Poland			Greece, Romania, Türkiye, United Arab Emirates	China, Colombia, Greece, Israel, Korea (Republic), Mexico, Romania, Saudi Arabia, Türkiye, Thailand, United Arab Emirates
Romania		Türkiye	Thailand, Türkiye	Colombia, Poland, Thailand, Türkiye
Saudi Arabia		Israel, Spain, United Arab Emirates	Australia, Greece, Israel, Italy, Japan, Korea (Republic), Poland, Singapore, Spain, United Arab Emirates, United Kingdom	Australia, Belgium, Canada, Colombia, Denmark, France, Greece, Israel, Italy, Japan, Korea (Republic), Mexico, Netherlands, Poland, Romania, Singapore, Spain, Sweden, Türkiye, United Arab Emirates, United Kingdom,
Singapore			Australia, Canada, Greece, Israel, Italy, Korea (Republic), Spain, Saudi Arabia, United Arab Emirates	Australia, Belgium, Brazil, Canada, Denmark, France, Germany, Greece, Israel, Italy, Japan, Korea (Republic), Netherlands, Poland, Romania, Saudi Arabia, Spain, Sweden, Türkiye, United Arab Emirates, United Kingdom
Spain		Italy, Saudi Arabia	Australia, Belgium, Canada, France, Greece, Israel, Italy, Japan, Korea (Republic), Poland, Saudi Arabia, United Arab Emirates, United Kingdom	Australia, Belgium, Canada, Colombia, Denmark, France, Germany, Greece, Israel, Italy, Japan, Korea (Republic), Mexico, Netherlands, Poland, Romania, Saudi Arabia, Singapore, Sweden, Türkiye, United Arab Emirates, United Kingdom
Sweden		Belgium, Denmark, France, Germany, Japan, Netherlands	Belgium, Denmark, France, Germany, Japan, Netherlands, United Kingdom	Belgium, Canada, Denmark, France, Germany, Italy, Japan, Netherlands, Spain, United Arab Emirates, United Kingdom

Thailand				Colombia, Türkiye
Türkiye		Romania	Romania, Thailand	Colombia, Poland, Romania, Thailand
United Arab Emirates			Greece, Israel, Italy, Korea (Republic), Poland, Saudi Arabia, Spain	China, Colombia, Greece, Israel, Italy, Korea (Republic), Mexico, Poland, Romania, Saudi Arabia, Singapore, Spain, Türkiye, United Kingdom
United Kingdom		Belgium, France, Japan	Belgium, Canada, Denmark, France, Germany, Italy, Japan, Netherlands, Spain, Sweden, United Arab Emirates	Australia, Belgium, Canada, Denmark, France, Germany, Italy, Japan, Netherlands, Poland, Romania, Saudi Arabia, Spain, Sweden, Türkiye, United Arab Emirates
United States of America			Denmark, Germany, Netherlands, Sweden	Australia, Belgium, Canada, Denmark, France, Germany, Italy, Japan, Netherlands, Spain, Sweden, United Arab Emirates, United Kingdom

Supplementary methodology references

1. The World Bank. UHC service coverage index. 2021. https://data.worldbank.org/indicator/SH.UHC.SRVS.CV.XD. Accessed 1 June 2021.
2. The World Bank. Domestic private health expenditure (% of current health expenditure). 2021. https://data.worldbank.org/indicator/SH.XPD.PVTD.CH.ZS. Accessed 1 June 2021.
3. The World Bank. Current health expenditure per capita, PPP (current international $). 2021. https://data.worldbank.org/indicator/SH.XPD.CHEX.PP.CD. Accessed 1 June 2021.
4. The World Bank. World Bank Data repository. 2019. https://data.worldbank.org/. Accessed 24 March 2022.
5. World Health Organization (WHO). The Global Health Observatory. 2019. https://www.who.int/data/gho. Accessed 24 March 2022.

Supplementary results

***Table S3:* Projected direct costs of diagnosed CKD and KRT in 2022 and 2027**

Country/region		Total direct costs of diagnosed CKD and KRT				
		2022		2027		Change in total cost 2022–2027, %
		Total cost US$, billion	Cost per capita, US$ per person*	Total cost US$, billion	Cost per capita, US$ per person*	
**Americas**	Brazil	**9·77**	45·38	**11·59**	52·42	18·61
	Canada	**7·38**	192·20	**8·09**	202·53	9·63
	Colombia	**1·76**	34·23	**2·19**	41·63	24·00
	Mexico	**5·97**	45·35	**6·38**	46·33	6·88
	USA†	**161·95**	483·70	**173·41**	503·96	7·08
**Europe**	Belgium	**2·84**	243·50	**2·86**	242·12	0·70
	Denmark	**0·48**	81·48	**0·53**	88·75	10·94
	France	**14·52**	221·39	**17·93**	270·29	23·47
	Germany	**12·34**	147·16	**13·81**	165·67	11·85
	Greece	**1·29**	124·77	**1·46**	145·42	13·59
	Hungary	**0·85**	88·46	**0·87**	92·16	2·47
	Italy	**5·94**	98·50	**6·08**	102·04	2·38
	Netherlands	**1·80**	104·59	**2·02**	115·98	11·97
	Poland	**1·41**	37·34	**1·73**	46·48	23·10
	Romania	**1·35**	70·73	**1·84**	99·01	36·75
	Spain	**7·15**	149·46	**8·14**	167·90	13·81
	Sweden	**1·25**	122·79	**1·26**	120·36	0·61
	Türkiye	**4·58**	53·51	**5·04**	57·55	10·12
	UK	**4·48**	65·43	**4·71**	67·48	5·04
**Asia-Pacific/ Middle East**	Australia	**2·35**	90·18	**2·66**	96·94	13·02
	China	**45·07**	31·11	**45·21**	30·93	0·33
	India†	**15·87**	11·28	**16·17**	11·01	1·90
	Israel	**1·55**	173·22	**1·53**	159·41	-1·16
	Japan	**33·76**	268·83	**38·31**	312·05	13·46
	Philippines	**1·93**	17·15	**2·17**	18·16	12·58
	Saudi Arabia	**6·50**	181·37	**8·71**	228·56	34·00
	Singapore	**1·07**	179·60	**1·29**	209·89	21·09
	South Korea	**6·90**	134·49	**8·45**	164·67	22·35
	Taiwan	**3·47**	145·38	**4·16**	173·20	19·68
	Thailand	**6·12**	87·26	**7·75**	110·05	26·68
	UAE Emirati	**0·31**	190·19	**0·38**	216·87	24·33
	**Sum**	**372·00**		**406·72**		
	UAE expatriate‡	0·03	3·68	0·06	7·18	100·00

CKD=chronic kidney disease. KRT=kidney replacement therapy. Note: values presented are rounded to 2 decimal places. There may be slight discrepancies in the total sum presented due to rounding. *Cost per capita represents the total cost divided by the entire general population. †In settings without a single public healthcare system, the model was adapted to use a commercial or equivalent framework, or a mixed funding model. For

example, in the case of the USA, costs were split into Medicare and commercial categories; for India, costs were split into four components: charitable, private, employment insurance, and public. ‡UAE expatriate population was modelled separately from the Emirati population owing to distinct population demographics requiring separate input data.

Table S4: **Projected direct costs of diagnosed CKD (pre-KRT) by CKD stage in 2022 and 2027.**

Country/region		Year	Direct cost, US$, billion				
			CKD stage (pre-KRT)				
			3a	3b	4	5	Sum
Americas	Brazil	2022	3·74	2·71	0·56	0·07	**7·08**
		2027	4·67	2·45	0·72	0·20	**8·04**
	Canada	2022	2·92	1·48	0·82	0·29	**5·51**
		2027	3·31	1·34	0·68	0·60	**5·93**
	Colombia	2022	0·55	0·18	0·07	0·01	**0·81**
		2027	0·84	0·15	0·06	0·02	**1·06**
	Mexico	2022	1·27	0·81	0·33	0·74	**3·14**
		2027	1·86	0·73	0·30	0·32	**3·22**
	USA*	2022	51·95	33·80	5·07	0·55	**91·37**
		2027	58·21	30·59	8·76	1·95	**99·51**
Europe	Belgium	2022	0·88	0·38	0·46	0·16	**1·88**
		2027	0·98	0·32	0·27	0·21	**1·77**
	Denmark	2022	0·16	0·06	0·03	0·004	**0·26**
		2027	0·19	0·04	0·02	0·01	**0·25**
	France	2022	3·02	1·08	1·05	0·00	**5·15**
		2027	4·05	0·90	0·91	0·00	**5·86**
	Germany	2022	3·67	1·21	0·48	0·14	**5·49**
		2027	4·88	1·03	0·42	0·26	**6·59**
	Greece	2022	0·19	0·04	0·05	0·01	**0·29**
		2027	0·23	0·03	0·03	0·02	**0·30**
	Hungary	2022	0·20	0·14	0·04	0·004	**0·38**
		2027	0·23	0·08	0·02	0·01	**0·34**
	Italy	2022	1·04	0·76	0·58	0·16	**2·54**
		2027	1·39	0·73	0·41	0·12	**2·64**
	Netherlands	2022	0·37	0·13	0·06	0·01	**0·58**
		2027	0·49	0·10	0·04	0·02	**0·65**
	Poland	2022	0·02	0·03	0·02	0·001	**0·07**
		2027	0·03	0·02	0·01	0·002	**0·06**
	Romania	2022	0·46	0·09	0·02	0·002	**0·57**
		2027	0·70	0·06	0·02	0·01	**0·79**
	Spain	2022	2·04	1·60	0·55	0·10	**4·29**
		2027	2·43	1·63	0·44	0·19	**4·68**
	Sweden	2022	0·24	0·30	0·18	0·01	**0·73**

		2027	0·32	0·21	0·10	0·02	**0·66**
	Türkiye	2022	1·06	0·81	0·69	0·14	**2·69**
		2027	1·54	0·52	0·66	0·10	**2·81**
	UK	2022	1·80	0·75	0·34	0·08	**2·97**
		2027	1·93	0·63	0·24	0·13	**2·95**
**Asia-Pacific/ Middle East**	Australia	2022	0·66	0·16	0·31	0·10	**1·23**
		2027	0·88	0·17	0·26	0·09	**1·41**
	China	2022	16·73	3·24	4·01	0·48	**24·46**
		2027	17·79	3·14	2·07	0·33	**23·33**
	India*	2022	8·37	3·01	0·87	1·43	**13·68**
		2027	11·30	1·90	0·50	0·25	**13·95**
	Israel	2022	0·42	0·28	0·20	0·07	**0·96**
		2027	0·51	0·21	0·11	0·07	**0·90**
	Japan	2022	6·71	5·74	3·50	0·23	**16·18**
		2027	10·44	3·68	4·50	0·69	**19·31**
	Philippines	2022	0·34	0·08	0·04	0·08	**0·54**
		2027	0·41	0·09	0·03	0·06	**0·58**
	Saudi Arabia	2022	2·40	1·26	0·74	0·80	**5·20**
		2027	3·25	1·55	0·87	0·84	**6·50**
	Singapore	2022	0·54	0·11	0·06	0·02	**0·71**
		2027	0·65	0·14	0·05	0·02	**0·86**
	South Korea	2022	0·80	0·21	0·16	0·04	**1·22**
		2027	1·37	0·24	0·22	0·11	**1·95**
	Taiwan	2022	0·60	0·15	0·21	0·01	**0·97**
		2027	1·01	0·17	0·26	0·03	**1·48**
	Thailand	2022	0·84	0·28	0·12	0·15	**1·39**
		2027	0·93	0·34	0·15	0·21	**1·63**
	UAE Emirati	2022	0·04	0·03	0·02	0·004	**0·09**
		2027	0·07	0·03	0·03	0·003	**0·12**
**Sum**		**2022**					**202·43**
		**2027**					**220·14**
	UAE expatriate†	*2022*	*0·02*	*0·01*	*0·002*	*0·002*	*0·03*
		2027	*0·04*	*0·01*	*0·004*	*0·001*	*0·05*

CKD=chronic kidney disease. KRT=kidney replacement therapy. Note: values presented are rounded to 2 decimal places. There may be slight discrepancies in the total sum presented due to rounding. *In settings without a single public healthcare system, the model was adapted to use a commercial or equivalent framework, or a mixed funding model. For example, in the case of the USA, costs were split into Medicare and commercial

categories; for India, costs were split into four components: charitable, private, employment insurance, and public. For these countries, distinct input data for each aspect of the funding models were used. [†]UAE expatriate population was modelled separately from the Emirati population owing to distinct population demographics requiring separate input data.

Table S5: **Projected direct costs of diagnosed CKD (pre-KRT) costs by age group in 2022 and 2027**

Country/region		18–34 years old					35–64 years old					65–110 years old				
		2022		2027			2022		2027			2022		2027		
		**Total cost, US$, billion**	**Cost per capita, US$, per person***	**Total cost, US$, billion**	**Cost per capita, US$ per person***	Change in total cost 2022–2027, %	**Total cost, US$, billion**	**Cost per capita, US$ per person***	**Total cost, US$, billion**	**Cost per capita, US$ per person***	Change in total cost 2022–2027, %	**Total cost, US$, billion**	**Cost per capita, US$ per person***	**Total cost, US$, billion**	**Cost per capita, US$ per person***	Change in total cost 2022–2027, %
**Americas**	Brazil	**0·17**	0·8	**0·26**	1·2	59·4	**1·91**	8·9	**2·76**	12·5	44·6	**5·01**	23·3	**5·02**	22·7	0·2
	Canada	**0·05**	1·3	**0·10**	2·6	100·5	**0·88**	23·0	**1·21**	30·3	37·2	**4·57**	119·1	**4·62**	115·6	0·9
	Colombia	**0·01**	0·3	**0·04**	0·8	187·8	**0·20**	3·9	**0·40**	7·6	97·6	**0·59**	11·5	**0·62**	11·9	5·2
	Mexico	**0·06**	0·46	**0·08**	0·6	27·5	**0·72**	5·5	**1·32**	9·6	84·1	**2·37**	18·0	**1·82**	13·2	-23·1
	USA†	**0·99**	3·0	**1·35**	3·9	36·2	**18·66**	55·7	**26·20**	76·1	40·4	**71·71**	214	**71·96**	209·1	0·4
**Europe**	Belgium	**0·01**	1·2	**0·03**	2·2	89·5	**0·38**	32·7	**0·43**	36·8	13·9	**1·49**	127·6	**1·31**	111·2	-11·8
	Denmark	**0·001**	0·3	**0·004**	0·7	191·7	**0·04**	6·6	**0·06**	9·6	46·6	**0·22**	37·3	**0·19**	32·6	-11·0
	France	**0·07**	1·0	**0·07**	1·1	10·6	**0·87**	13·2	**1·43**	21·5	64·9	**4·22**	64·3	**4·36**	65·7	3·4
	Germany	**0·22**	2·6	**0·29**	3·5	33·6	**1·07**	12·7	**2·38**	28·6	122·9	**4·20**	50·1	**3·92**	47·0	-6·7
	Greece	**0·001**	0·1	**0·004**	0·4	166·5	**0·042**	4·1	**0·08**	7·7	83·6	**0·25**	23·8	**0·22**	22·3	-8·9
	Hungary	**0·002**	0·2	**0·01**	0·5	183·1	**0·05**	5·3	**0·08**	8·1	49·5	**0·33**	34·3	**0·26**	27·5	-21·0
	Italy	**–**	–	**–**	–	–	**0·29**	4·8	**0·41**	6·9	42·8	**2·25**	37·4	**2·23**	37·5	-0·8
	Netherlands	**0·004**	0·2	**0·01**	0·7	189·5	**0·09**	7·1	**0·16**	9·4	75·3	**0·48**	27·6	**0·47**	27·1	-1·5
	Poland	**0·0003**	0·01	**0·001**	0·03	260·9	**0·01**	0·2	**0·01**	0·3	46·7	**0·06**	1·7	**0·05**	1·3	-23·7
	Romania	**0·02**	1·0	**0·02**	1·0	1·9	**0·19**	10·2	**0·29**	15·8	50·6	**0·36**	18·7	**0·48**	25·8	34·6
	Spain	**0·03**	0·7	**0·06**	1·2	79·3	**0·65**	13·6	**1·07**	22·1	64·9	**3·61**	75·33	**3·56**	73·3	-1·4
	Sweden	**0·01**	0·5	**0·01**	0·7	36·4	**0·08**	8·0	**0·13**	12·7	62·6	**0·64**	62·8	**0·52**	49·5	-19·1
	Türkiye	**0·04**	0·4	**0·05**	0·6	35·7	**0·58**	6·8	**0·95**	10·9	64·2	**2·07**	24·2	**1·81**	20·6	-12·7
	UK	**0·03**	0·4	**0·06**	0·8	87·8	**0·48**	7·0	**0·68**	9·7	40·8	**2·46**	35·9	**2·21**	31·7	-10·1
**Asia-Pacific/ Middle East**	Australia	**0·01**	0·3	**0·01**	0·5	42·0	**0·17**	6·4	**0·32**	11·6	90·8	**1·06**	40·5	**1·08**	39·3	2·0
	China	**0·14**	0·1	**0·29**	0·2	107·1	**3·43**	2·4	**5·48**	3·8	59·7	**20·89**	14·4	**17·56**	12·0	-16·0
	India†	**0·23**	0·2	**0·45**	0·3	96·0	**2·73**	1·9	**4·64**	3·2	69·8	**10·72**	7·6	**8·86**	6·0	-17·3
	Israel	**0·01**	1·4	**0·02**	2·0	57·0	**0·13**	14·1	**0·20**	21·1	61·0	**0·82**	92·4	**0·68**	70·6	-17·9

	Japan	**0·22**	1·7	**0·40**	3·3	83·3	**2·12**	16·9	**4·70**	38·3	121·7	**13·84**	110·2	**14·20**	115·7	2·6
	Philippines	**0·01**	0·1	**0·02**	0·2	72·7	**0·14**	1·3	**0·22**	1·8	52·7	**0·38**	3·4	**0·34**	2·9	-10·8
	Saudi Arabia	**0·96**	26·8	**0·80**	20·9	-16·9	**3·37**	94·1	**4·56**	119·5	35·1	**0·84**	23·4	**1·15**	30·1	36·8
	Singapore	**0·01**	1·5	**0·02**	2·5	70·5	**0·20**	33·1	**0·25**	40·4	26·3	**0·51**	85·6	**0·59**	96·3	16·5
	South Korea	**0·04**	0·7	**0·11**	2·1	181·2	**0·42**	8·1	**0·48**	9·4	15·5	**0·72**	14·0	**1·23**	24·0	71·4
	Taiwan	**0·03**	1·3	**0·08**	3·4	168·3	**0·33**	13·7	**0·37**	15·4	12·5	**0·58**	24·2	**0·92**	38·4	59·1
	Thailand	**0·01**	0·1	**0·01**	0·1	44·5	**0·61**	8·7	**0·68**	9·7	12·4	**0·77**	11·0	**0·93**	13·3	21·0
	UAE Emirati	**0·02**	10·9	**0·02**	9·0	-9·7	**0·06**	37·2	**0·07**	40·8	19·7	**0·02**	10·6	**0·03**	19·3	98·2
	UAE expatriate‡	***0·002***	*0·2*	***0·01***	*0·6*	*204·6*	***0·01***	*1·7*	***0·03***	*3·9*	*138·5*	***0·01***	*1·6*	***0·02***	*1·8*	*14·4*
**Sum**		**3·39**		**4·66**			**40·89**		**62·02**			**158·03**		**153·22**		
**Mean**		**0·11**		**0·15**			**1·28**		**1·94**			**4·94**		**4·79**		

CKD=chronic kidney disease. KRT=kidney replacement therapy. Note: values presented are rounded. There may be slight discrepancies in the total sum presented due to rounding.

*Cost per capita represents the total cost divided by the entire general population. †In settings without a single public healthcare system, the model was adapted to use a commercial or equivalent framework, or a mixed funding model. For example, in the case of the USA, costs were split into Medicare and commercial categories; for India, costs were split into four components: charitable, private, employment insurance, and public. For these countries, distinct input data for each aspect of the funding models were used. ‡UAE expatriate population was modelled separately from the Emirati population owing to distinct population demographics requiring separate input data.

Table S6: **Projected direct costs of KRT by modality in 2022 and 2027**

Country/Region		Haemodialysis					Peritoneal dialysis					Kidney transplant incidence					Kidney transplant maintenance					Sum of modalities	
		2022		2027			2022		2027			2022		2027			2022		2027				
		Total cost, US$, billions	Cost per capita, US$	Total cost, US$, billions	Cost per capita, US$	Change in total cost 2022–2027, %	Total cost, US$, billions	Cost per capita, US$	Total cost, US$, billions	Cost per capita, US$	Change in total cost 2022–2027, %	Total cost, US$, billions	Cost per capita, US$	Total cost, US$, billions	Cost per capita, US$	Change in total cost 2022–2027, %	Total cost, US$, billions	Cost per capita, US$	Total cost, US$, billions	Cost per capita, US$	Change in total cost 2022–2027, %	2022	2027
**Americas**	Brazil	**2·18**	10·1	**2·82**	12·8	29·8	**0·18**	0·8	**0·29**	1·3	61·6	**0·04**	0·2	**0·13**	0·6	242·6	**0·30**	1·4	**0·31**	1·4	3·1	**2·69**	**3·55**
	Canada	**1·26**	32·7	**1·49**	37·3	18·5	**0·19**	5·0	**0·24**	6·1	24·9	**0·06**	1·6	**0·06**	1·5	1·0	**0·36**	9·4	**0·37**	9·2	2·1	**1·87**	**2·16**
	Colombia	**0·65**	12·6	**0·73**	14·0	13·2	**0·24**	4·6	**0·29**	5·5	22·0	**0·02**	0·3	**0·05**	0·9	159·6	**0·05**	1·0	**0·05**	1·0	7·0	**0·96**	**1·12**
	Mexico	**1·35**	10·3	**1·67**	12·1	23·4	**0·76**	5·8	**0·81**	5·9	5·8	**0·15**	1·1	**0·10**	0·7	-29·7	**0·56**	4·3	**0·58**	4·2	3·5	**2·82**	**3·16**
	USA	**52·85**	157·8	**55·04**	159·9	4·1	**5·25**	15·7	**5·84**	17·0	11·2	**0·40**	1·2	**0·72**	2·1	79·8	**12·08**	36·1	**12·31**	35·8	1·9	**70·58**	**73·90**
**Europe**	Belgium	**0·82**	70·4	**0·92**	78·1	12·3	**0·05**	3·9	**0·05**	4·3	11·3	**0·02**	1·3	**0·03**	2·8	113·2	**0·07**	6·3	**0·08**	6·7	8·6	**0·96**	**1·09**
	Denmark	**0·15**	25·0	**0·18**	30·4	23·9	**0·02**	4·1	**0·03**	5·7	41·6	**0·003**	0·6	**0·01**	1·9	246·0	**0·04**	7·6	**0·05**	7·8	5·2	**0·22**	**0·27**
	France	**7·33**	111·7	**9·45**	142·5	29·0	**0·34**	5·1	**0·47**	7·0	38·3	**0·48**	7·4	**0·78**	11·8	61·5	**1·23**	18·7	**1·37**	20·7	11·8	**9·37**	**12·07**
	Germany	**6·11**	72·8	**6·41**	76·9	5·0	**0·31**	3·7	**0·33**	3·9	6·2	**0·03**	0·3	**0·06**	0·8	125·1	**0·41**	4·9	**0·42**	5·0	0·9	**6·86**	**7·22**
	Greece	**0·88**	85·4	**1·02**	101·5	15·8	**0·07**	6·4	**0·08**	7·6	15·9	**0·02**	1·8	**0·03**	3·0	61·9	**0·03**	3·2	**0·03**	3·1	-5·5	**1·00**	**1·16**
	Hungary	**0·23**	23·5	**0·24**	25·1	4·9	**0·02**	2·5	**0·03**	2·7	5·5	**0·004**	0·4	**0·04**	4·1	921·0	**0·21**	22·3	**0·23**	24·2	6·8	**0·47**	**0·53**
	Italy	**2·34**	38·8	**2·40**	40·3	2·6	**0·30**	5·0	**0·32**	5·4	6·1	**0·25**	4·2	**0·17**	2·8	-34·0	**0·51**	8·4	**0·55**	9·2	8·3	**3·40**	**3·43**
	Netherlands	**0·71**	41·2	**0·81**	46·8	14·8	**0·14**	8·0	**0·17**	9·5	19·2	**0·01**	0·6	**0·02**	1·2	108·6	**0·37**	21·3	**0·37**	21·3	0·8	**1·22**	**1·37**
	Poland	**1·14**	30·3	**1·43**	38·3	24·9	**0·06**	1·7	**0·09**	2·3	34·8	**0·01**	0·2	**0·03**	0·8	356·0	**0·12**	3·2	**0·13**	3·5	5·8	**1·34**	**1·67**
	Romania	**0·74**	39·0	**0·88**	47·4	18·7	**0·01**	0·6	**0·01**	0·7	13·7	**0·01**	0·8	**0·15**	7·8	880·5	**0·01**	0·4	**0·01**	0·4	10·7	**0·78**	**1·05**
	Spain	**2·19**	45·8	**2·62**	54·0	19·3	**0·28**	5·9	**0·37**	7·7	31·3	**0·01**	0·2	**0·07**	1·5	503·5	**0·38**	7·9	**0·40**	8·2	4·9	**2·87**	**3·46**
	Sweden	**0·34**	33·6	**0·38**	36·4	11·3	**0·06**	5·9	**0·07**	7·1	21·9	**0·03**	2·5	**0·04**	3·6	48·5	**0·10**	9·4	**0·11**	10·4	13·2	**0·53**	**0·60**
	Türkiye	**1·42**	16·6	**1·71**	19·6	20·4	**0·17**	2·0	**0·25**	2·8	45·9	**0·11**	1·3	**0·07**	0·8	-41·1	**0·18**	2·1	**0·20**	2·3	9·1	**1·89**	**2·23**
	UK	**0·95**	13·8	**1·12**	16·1	18·1	**0·13**	1·9	**0·20**	2·8	53·9	**0·04**	0·6	**0·05**	0·7	13·9	**0·39**	5·7	**0·40**	5·7	1·1	**1·51**	**1·76**
**Asia-Pacific/**	Australia	**0·82**	31·5	**0·93**	33·8	12·8	**0·12**	4·7	**0·15**	5·3	18·9	**0·02**	0·9	**0·02**	0·8	-11·5	**0·15**	5·9	**0·16**	5·8	3·1	**1·12**	**1·25**
	China	**17·24**	11·9	**18·42**	12·6	6·8	**1·46**	1·0	**1·59**	1·1	8·9	**0·07**	0·0	**0·03**	0·0	-55·7	**1·83**	1·3	**1·84**	1·3	0·4	**20·60**	**21·88**

**Middle East**	India	**1·00**	0·7	**0·99**	0·7	-0·9	**0·07**	0·1	**0·08**	0·1	5·2	**0·16**	0·1	**0·14**	0·1	-13·4	**0·95**	0·7	**1·01**	0·7	6·0	**2·19**	**2·22**
	Israel	**0·52**	58·2	**0·56**	58·2	7·4	**0·04**	4·3	**0·04**	4·2	4·6	**0·02**	1·9	**0·02**	1·6	-7·6	**0·01**	1·0	**0·01**	1·3	39·1	**0·58**	**0·63**
	Japan	**17·08**	136·0	**18·42**	150·1	7·8	**0·36**	2·9	**0·41**	3·4	15·0	**0·01**	0·04	**0·02**	0·2	303·9	**0·13**	1·0	**0·14**	1·2	8·4	**17·58**	**19·00**
	Philippines	**1·32**	11·7	**1·52**	12·7	15·0	**0·03**	0·3	**0·04**	0·3	22·0	**0·02**	0·2	**0·02**	0·1	-27·3	**0·02**	0·1	**0·02**	0·2	12·5	**1·39**	**1·59**
	KSA	**0·95**	26·6	**1·55**	40·6	62·2	**0·11**	3·1	**0·21**	5·6	93·6	**0·16**	4·3	**0·18**	4·8	17·3	**0·08**	2·2	**0·27**	7·0	229·5	**1·30**	**2·21**
	Singapore	**0·28**	47·9	**0·35**	56·1	21·2	**0·03**	5·6	**0·05**	7·4	36·5	**0·01**	1·2	**0·02**	2·5	111·1	**0·03**	4·5	**0·03**	4·8	9·2	**0·35**	**0·44**
	South Korea	**4·35**	84·8	**4·99**	97·4	14·7	**0·20**	4·0	**0·22**	4·4	10·0	**0·03**	0·7	**0·10**	1·9	190·0	**1·10**	21·3	**1·18**	23·1	7·9	**5·69**	**6·50**
	Taiwan	**2·31**	96·6	**2·47**	102·9	7·0	**0·16**	6·7	**0·17**	7·3	8·9	**0·0003**	0·0	**0·001**	0·0	163·0	**0·03**	1·3	**0·03**	1·4	3·5	**2·50**	**2·68**
	Thailand	**3·97**	56·6	**5·03**	71·5	26·8	**0·27**	3·9	**0·34**	4·8	23·7	**0·18**	2·5	**0·22**	3·2	27·3	**0·32**	4·5	**0·53**	7·6	68·2	**4·73**	**6·12**
	UAE Emitrati	**0·17**	108·6	**0·21**	120·2	20·7	**0·01**	6·7	**0·01**	7·0	14·7	**0·02**	10·0	**0·02**	10·4	13·8	**0·01**	5·9	**0·02**	10·0	83·5	**0·21**	**0·26**
	UAE expatriate	**0·004**	0·5	**0·01**	1·1	101·1	**0·0002**	0·024	**0·001**	0·1	270·7	**0·001**	0·2	**0·001**	0·1	-59·1	**0·0003**	0·039	**0·0005**	0·1	54·1	**0·01**	**0·01**
**Sum**		**133·7**		**146·8**			**11·5**		**13·2**			**2·4**		**3·4**			**22·1**		**23·2**				
**Mean**		**4·2**		**4·6**			**0·4**		**0·4**			**0·1**		**0·1**			**0·7**		**0·7**				

CKD=chronic kidney disease. KRT=kidney replacement therapy. Note: values presented are rounded. There may be slight discrepancies in the total sum presented due to rounding. *Cost per capita represents the total cost divided by the entire general population. †In settings without a single public healthcare system, the model was adapted to use a commercial or equivalent framework, or a mixed funding model. For example, in the case of the USA, costs were split into Medicare and commercial categories; for India, costs were split into four components: charitable, private, employment insurance, and public. For these countries, distinct input data for each aspect of the funding models were used. ‡UAE expatriate population was modelled separately from the Emirati population owing to distinct population demographics requiring separate input data.

Table S7: **CKD and KRT costs as a proportion of national annual healthcare expenditure in 2022 and 2027**

Country/region		Year	Proportion of national annual healthcare expenditure* (%)							
			CKD stage				KRT			
			3a	3b	4	5	Haemodialysis	Peritoneal dialysis	Transplant	Sum
Americas	Brazil	2022	8·59	6·23	1·28	0·17	5·00	0·42	0·77	**22·44**
		2027	10·72	5·64	1·65	0·45	6·49	0·68	0·99	**26·62**
	Canada	2022	1·86	0·94	0·52	0·19	0·80	0·12	0·27	**4·71**
		2027	2·12	0·86	0·43	0·38	0·95	0·15	0·27	**5·16**
	Colombia	2022	1·16	0·39	0·15	0·01	1·37	0·50	0·14	3·72
		2027	1·76	0·32	0·12	0·04	1·55	0·61	0·21	4·61
	Mexico	2022	1·87	1·19	0·49	1·09	2·00	1·12	1·05	**8·81**
		2027	2·75	1·08	0·44	0·48	2·46	1·19	1·01	**9·42**
	USA†	2022	0·97	0·78	0·12	0·01	1·38	0·14	0·33	**3·72**
		2027	0·98	0·68	0·18	0·04	1·44	0·15	0·34	**3·82**
Europe	Belgium	2022	1·56	0·67	0·81	0·28	1·45	0·08	0·16	**5·03**
		2027	1·73	0·56	0·47	0·38	1·63	0·09	0·20	**5·06**
	Denmark	2022	0·55	0·21	0·09	0·01	0·49	0·08	0·16	**1·60**
		2027	0·63	0·15	0·05	0·03	0·61	0·11	0·19	**1·77**
	France	2022	1·10	0·39	0·38	0·00	2·67	0·12	0·62	**5·29**
		2027	1·48	0·33	0·33	0·00	3·45	0·17	0·78	**6·54**
	Germany	2022	0·84	0·28	0·11	0·03	1·39	0·07	0·10	**2·81**
		2027	1·11	0·23	0·10	0·06	1·46	0·07	0·11	**3·15**
	Greece	2022	0·54	0·13	0·14	0·02	2·51	0·19	0·15	**3·67**
		2027	0·65	0·10	0·08	0·04	2·91	0·22	0·17	**4·17**
	Hungary	2022	1·28	0·86	0·23	0·02	1·41	0·15	1·36	**5·30**
		2027	1·45	0·53	0·11	0·04	1·48	0·16	1·67	**5·43**
	Italy	2022	0·54	0·39	0·30	0·08	1·22	0·16	0·39	**3·09**
		2027	0·72	0·38	0·21	0·06	1·25	0·17	0·37	**3·16**
	Netherlands	2022	0·36	0·13	0·06	0·01	0·68	0·13	0·36	**1·72**
		2027	0·47	0·09	0·04	0·02	0·78	0·16	0·37	**1·93**
	Poland	2022	0·04	0·04	0·03	0·00	1·70	0·10	0·19	**2·09**
		2027	0·05	0·03	0·02	0·00	2·12	0·13	0·23	**2·58**
	Romania	2022	2·13	0·41	0·10	0·01	3·44	0·05	0·10	**6·24**
		2027	3·24	0·29	0·09	0·05	4·09	0·06	0·71	**8·53**
	Spain	2022	1·57	1·23	0·43	0·08	1·69	0·22	0·30	**5·52**
		2027	1·88	1·26	0·34	0·14	2·02	0·29	0·36	**6·29**
	Sweden	2022	0·38	0·49	0·29	0·01	0·55	0·10	0·20	**2·02**

		2027	0·51	0·34	0·17	0·04	0·61	0·12	0·24	**2·03**
	Türkiye	2022	1·33	1·02	0·86	0·17	1·79	0·22	0·37	**5·76**
		2027	1·94	0·65	0·82	0·13	2·16	0·31	0·33	**6·34**
	UK	2022	0·81	0·34	0·15	0·04	0·43	0·06	0·20	**2·02**
		2027	0·87	0·29	0·11	0·06	0·50	0·09	0·20	**2·12**
**Asia-Pacific/ Middle East**	Australia	2022	0·70	0·17	0·33	0·10	0·87	0·13	0·19	**2·49**
		2027	0·93	0·18	0·28	0·09	0·98	0·15	0·19	**2·81**
	China	2022	1·21	0·23	0·29	0·04	1·25	0·11	0·14	**3·27**
		2027	1·29	0·23	0·15	0·02	1·34	0·12	0·14	**3·28**
	India†	2022	8·44	3·04	0·88	1·44	1·01	0·07	1·13	**16·00**
		2027	11·40	1·92	0·50	0·25	1·00	0·08	1·16	**16·31**
	Israel	2022	1·24	0·82	0·60	0·19	1·54	0·11	0·08	**4·57**
		2027	1·51	0·63	0·31	0·22	1·65	0·12	0·08	**4·52**
	Japan	2022	1·46	1·25	0·76	0·05	3·72	0·08	0·03	**7·36**
		2027	2·28	0·80	0·98	0·15	4·02	0·09	0·04	**8·35**
	Philippines	2022	0·81	0·18	0·11	0·20	3·20	0·07	0·10	**4·67**
		2027	0·99	0·21	0·07	0·14	3·67	0·08	0·09	**5·25**
	Saudi Arabia	2022	2·41	1·26	0·75	0·81	0·96	0·11	0·24	**6·53**
		2027	3·26	1·56	0·88	0·84	1·55	0·21	0·45	**8·76**
	Singapore	2022	1·87	0·37	0·19	0·06	0·99	0·12	0·12	**3·72**
		2027	2·26	0·49	0·16	0·07	1·20	0·16	0·16	**4·50**
	South Korea	2022	0·91	0·24	0·19	0·05	4·95	0·23	1·28	**7·85**
		2027	1·56	0·27	0·25	0·13	5·68	0·26	1·46	**9·60**
	Taiwan	2022	1·37	0·35	0·47	0·03	5·27	0·37	0·07	**7·93**
		2027	2·31	0·39	0·60	0·08	5·64	0·40	0·08	**9·49**
	Thailand	2022	1·68	0·56	0·24	0·29	7·91	0·54	0·98	**12·19**
		2027	1·85	0·67	0·31	0·42	10·03	0·67	1·51	**15·44**
	UAE Emirati	2022	0·17	0·11	0·10	0·02	0·74	0·05	0·11	**1·29**
		2027	0·28	0·12	0·11	0·01	0·89	0·05	0·15	**1·60**
	UAE expatriate‡	*2022*	0·07	0·03	0·01	0·01	0·02	0·001	0·01	**0·15**
		2027	0·18	0·03	0·02	0·003	0·04	0·003	0·004	**0·27**

CKD=chronic kidney disease. KRT=kidney replacement therapy. Note: values presented are rounded to 2 decimal places. There may be slight discrepancies in the total sum presented due to rounding. *The annual national healthcare budget represents 100% for each table row. †In settings without a single public healthcare system, the model was adapted to use a commercial or equivalent framework, or a mixed funding model. For example, in the case of the USA, costs were split into Medicare and commercial categories; for India, costs were split into four components: charitable, private, employment insurance, and public. For these countries, distinct input data for each aspect of the funding models were used. ‡UAE expatriate population was modelled separately from the Emirati population owing to distinct population demographics requiring separate input data.

Table S8: **External validation of economic burden of disease outputs.**
Please note that not all countries/regions and/or outputs are listed owing to a paucity of published data for comparison

**Cost output**	**External validation reference**	**External validation value**	***Inside CKD* 2022 value**	**Notes**
**Australia**				
CKD costs (pre-KRT)	(1) Australian Institute of Health and Welfare, 2005; (139) Tucker et al., 2014	US$0·949 billion	US$1·23 billion	Health expenditure attributed to CKD in 2000–2001 was US$647 million
Projected increase in CKD costs	(4) Tucker et al., 2014	37%	14·26%	Increase over time for Tucker et al., 2012–2020 vs *Inside CKD* 2022–2027
KRT costs as a proportion of national annual healthcare expenditure	(5) Van Der Tol et al., 2019	0·84%	1·19%	Percentage of public health expenditure spent on dialysis in 2016 vs 2022
**Belgium**				
KRT costs as a proportion of national annual healthcare expenditure	(5) Van Der Tol et al., 2019	1·36%	1·69%	Percentage of public health expenditure spent on dialysis in 2016 vs 2022
**Brazil**				
Projected increase in CKD costs	(6) Alcalde et al., 2018	10·95%	13·55% (pre-KRT)	Increase over time for Alcalde et al., 2013–2015 vs *Inside CKD* 2022–2027
**Canada**				
KRT costs as a proportion of national annual healthcare expenditure	(7) Zelmer et al., 2007	1·3%	1·20%	

**Colombia**				
KRT costs as a proportion of national annual healthcare expenditure	(5) Van Der Tol et al., 2019	1·46%	2·01%	Percentage of public health expenditure spent on dialysis in 2016 vs 2022
**Germany**				
KRT costs as a proportion of national annual healthcare expenditure	(2) Gandjour et al., 2020	1·6%	1·56%	
**Greece**				
KRT costs as a proportion of national annual healthcare expenditure	(8) Kaitelidou et al., 2004	2%	2·51%	Percentage of public health expenditure spent on haemodialysis
**India**				
KRT costs as a proportion of national annual healthcare expenditure	(5) Van Der Tol et al., 2019	0·71%	1·08%	Percentage of public health expenditure spent on dialysis in 2016 vs 2022
**Israel**				
KRT costs as a proportion of national annual healthcare expenditure	(5) Van Der Tol et al., 2019	2·35%	1·65%	Percentage of public health expenditure spent on dialysis in 2016 vs 2022
**Italy**				
Total CKD costs (pre-KRT)	(9) Turchetti et al., 2016	€1·81 billion	€1·65 billion (US$2·54 billion)	
KRT costs as a proportion of national annual	(10) Pontoriero et al., 2007;	1·8% (kidney failure); 0·97% (dialysis)	1·32% (CKD stages 3a–5);	Percentage of public health expenditure

healthcare expenditure	(5) Van Der Tol et al., 2019		1·37% (dialysis)	spent on dialysis in 2016 vs 2022
**Japan**				
Total CKD costs (pre-KRT)	(3) Ministry of Health, Labour and Welfare, 2016	￥1·546 trillion	￥1·556 trillion (US$16·18 billion)	Cost of kidney failure. Under Japanese disease classification, the term 'kidney failure' is used for kidney disease
KRT costs as a proportion of national annual healthcare expenditure	(5) Van Der Tol et al., 2019	2·83%	3·80%	Percentage of public health expenditure spent on dialysis in 2016 vs 2022
**Mexico**				
KRT costs as a proportion of national annual healthcare expenditure	(5) Van Der Tol et al., 2019	3·07%	3·12%	Percentage of public health expenditure spent on dialysis in 2016 vs 2022
**Netherlands**				
KRT costs as a proportion of national annual healthcare expenditure	(5) Van Der Tol et al., 2019	0·78%	0·81%	Percentage of public health expenditure spent on dialysis in 2016 vs 2022
**Philippines**				
KRT costs as a proportion of national annual healthcare expenditure	(11) Teerawattananon et al., 2020; (5) Van Der Tol et al., 2019	7·7%; 2·74%	3·36%; 3·26%	KRT spending as a proportion of national healthcare expenditure; Percentage of public health expenditure spent on dialysis in 2016 vs 2022
**Poland**				
KRT costs as a proportion of national annual	(12) Gellert et al., 2018	1·1%	1·98%	Percentage of public health expenditure

healthcare expenditure				spent on KRT in 2016 vs 2022
**Saudi Arabia**				
KRT costs as a proportion of national annual healthcare expenditure	(5) Van Der Tol et al., 2019	2·30%	1·07%	Percentage of public health expenditure spent on dialysis in 2016 vs 2022
**South Korea**				
Total CKD costs (pre-KRT)	(13) Lee et al., 2018	US$1·51 billion	US$1·22 billion	National Health Insurance Corporation calculations
Total KRT costs	(15) Bikbov et al., 2019	US$3·3 billion	US$5·69 billion	
**Spain**				
Total KRT costs	(16) Villa et al., 2011	€1·407 billion	€1·72 billion (US$2·87 billion)	Total budgetary impact of the Spanish KRT programme amounted to ~€1·407 billion (excluding indirect costs), and €1·829 billion (including indirect costs). Perspective of the Public Administration
KRT costs as a proportion of national annual healthcare expenditure	(17) Ortiz et al., 2019	2–5%	2·21%	
**Taiwan**				
Total CKD costs (pre-KRT)	(18) Taipei Times, 2020	US$1·8 billion	US$0·97 billion	Based on 2019 data (US$1·8 billion). Not inflated
**Türkiye**				
KRT costs as a proportion of national	(19) Erek et al., 2004;	5·50%;<br>1·41%	2·37%;<br>2·01%	Percentage of public health expenditure spent on KRT in

annual healthcare expenditure	(5) Van Der Tol et al., 2019			2000 vs 2022; Percentage of public health expenditure spent on dialysis in 2016 vs 2022
**UK**				
Total CKD costs (pre-KRT)	(20) Kerr et al., 2012	£1·44–1·45 billion	£2·01 billion (US$2·97 billion)	
KRT costs as a proportion of national annual healthcare expenditure	(5) Van Der Tol et al., 2019; (20) Kerr et al., 2012	0·39–0·65%	0·68%	Kerr et al., 2012: *"The cost of CKD to the English NHS in 2009-10 is estimated at £1·44 to £1·45 billion, which is approximately 1·3% of all NHS spending in that year. More than half this sum was spent on RRT…"*
**USA**				
Total CKD costs (pre-KRT)	(21) Centers for Disease Control and Prevention, 2018	US$86·76 billion	US$91·37 billion	
KRT costs as a proportion of national annual healthcare expenditure	(5) Van Der Tol et al., 2019	1·10%	1·52%	Dialysis only

CKD=chronic kidney disease. KRT=kidney replacement therapy. NHS=National Health Service. RRT=renal replacement therapy.

Supplementary results references

1. Australian Institute of Health and Welfare. Health expenditure. 2022. https://www.aihw.gov.au/reports/health-welfare-expenditure/health-expenditure (accessed Oct 13, 2022).
2. Gandjour A, Armsen W, Wehmeyer W et al. Costs of patients with chronic kidney disease in Germany. *PLoS One* 2020; **15**: e0231375.
3. Ministry of Health, Labour and Welfare of Japan. Estimates of national medical care expenditure. 2019. https://www.mhlw.go.jp/stf/shingi2/0000196043_00003.html (accessed Oct 13, 2022).
4. Tucker PS, Kingsley MI, Morton RH, Scanlan AT, Dalbo VJ. The increasing financial impact of chronic kidney disease in Australia. *Int J Nephrol* 2014: 120537.
5. van der Tol A, Lameire N, Morton RL, Van Biesen W, Vanholder R. An International Analysis of Dialysis Services Reimbursement. *Clin J Am Soc Nephrol* 2019; **14**: 84–93.
6. Alcalde PR, Kirsztajn GM. Expenses of the Brazilian Public Healthcare System with chronic kidney disease. *Braz J Nephrol* 2018; **40**: 122–9.
7. Zelmer JL. The economic burden of end-stage renal disease in Canada. *Kidney Int* 2007; **72**: 1122–9.
8. Kaitelidou D, Ziroyiannis PN, Maniadakis N, Liaropoulos L, Theodorou M. The socioeconomic impact of hemodialysis. *Hippokratia* 2004; **8**: 81–7.
9. Turchetti G, Bellelli S, Amato M, et al. The social cost of chronic kidney disease in Italy. *Eur J Health Econ* 2017; **18**: 847–58.
10. Pontoriero G, Pozzoni P, Vecchio LD, Locatelli F. International Study of Health Care Organization and Financing for renal replacement therapy in Italy: an evolving reality. *Int J Health Care Finance Econ* 2007; **7**: 201–15.
11. Teerawattananon Y, Dabak SV, Khoe LC, Bayani DBS, Isaranuwatchai W. To include or not include: renal dialysis policy in the era of universal health coverage. *BMJ* 2020; **368**: m82.
12. Gellert R. Opieka koordynowana nad pacjentem z przewlekłą chorobą nerek. 2018. http://www.izbamedpol.pl/wp-content/uploads/2019/03/opieka_koordynowana_raport_2019.pdf (accessed July 1, 2022). [Polish].
13. Lee HS, Ju Y-S, Song YR, et al. Current treatment status and medical costs for hemodialysis vascular access based on analysis of the Korean Health Insurance Database. *Korean J Intern Med* 2018; **33**: 1160–8.
14. Sun-Min K and Yong-Ik K. 2020 National health insurance statistical yearbook. Published October 2021 by Health Insurance Review & Assessment Service and National Health Insurance Service. https://www.hira.or.kr/bbsDummy.do?pgmid=HIRAJ030000007001&brdScnBltNo=4&brdBltNo=3 (accessed August 31 2023).
15. Bikbov B, Purcell CA, Levey AS, et al. Global, regional, and national burden of chronic kidney disease, 1990–2017: a systematic analysis for the Global Burden of Disease Study 2017. *Lancet* 2020; **395**: 709–33.
16. Villa G, Rodríguez-Carmonaet A, Fernández-Ortiz L, et al. *Nephrol Dial Transplant* 2011; **26**: 3709–14.
17. Ortiz A, Sanchez-Niño MD, Crespo-Barrio M, et al. The Spanish Society of Nephrology (SENEFRO) commentary to the Spain GBD 2016 report: Keeping chronic kidney disease out of sight of health authorities will only magnify the problem. *Nefrología* 2019; **39**: 29–34.
18. Taipei Times. Chronic kidney disease costs nation NT$53.3bn. 2020. https://www.taipeitimes.com/News/taiwan/archives/2020/07/13/2003739828 (accessed September 13, 2021).
19. Erek E, Sever MS, Akoglu E, et al. Cost of renal replacement therapy in Turkey. *Nephrology (Carlton)* 2004; **9**: 33–8.
20. Kerr M, Bray B, Medcalf J, O'Donoghue DJ, Matthews B. Estimating the financial cost of chronic kidney disease to the NHS in England. *Nephrol Dial Transplant* 2012;**27**: iii73–iii80.

21. Centers for Disease Control and Prevention. Chronic kidney disease basics. https://www.cdc.gov/kidneydisease/basics.html (accessed Sep 10, 2021).
